# Partial compartmentalisation of HIV-1 subtype C between lymph nodes, peripheral blood mononuclear cells and plasma

**DOI:** 10.1016/j.virol.2023.03.011

**Published:** 2023-05

**Authors:** Neschika Jeewanraj, Tawanda Mandizvo, Takalani Mulaudzi, Nombali Gumede, Zaza Ndhlovu, Thumbi Ndung'u, Kamini Gounder, Jaclyn Mann

**Affiliations:** a HIV Pathogenesis Programme, Doris Duke Medical Research Institute, Nelson R. Mandela School of Medicine, University of KwaZulu-Natal, Durban, South Africa; b Africa Health Research Institute, Durban, South Africa; c Ragon Institute of Massachusetts General Hospital, Massachusetts Institute of Technology and Harvard University, Cambridge, MA, USA; d Division of Infection and Immunity, University College London, London, United Kingdom

**Keywords:** HIV-1, Envelope, Gag, Compartmentalisation, Single genome amplification, Lymph node, Peripheral blood mononuclear cells, Plasma

## Abstract

HIV-1 compartmentalisation is likely to have important implications for a preventative vaccine as well as eradication strategies. We genetically characterised HIV-1 subtype C variants in lymph nodes, peripheral blood mononuclear cells and plasma of six antiretroviral (ART) naïve individuals and four individuals on ART. Full-length *env* (n = 171) and *gag* (n = 250) sequences were generated from participants using single genome amplification. Phylogenetic relatedness of sequences was assessed, and compartmentalisation was determined using both distance and tree-based methods implemented in HyPhy. Additionally, potential associations between compartmentalisation and immune escape mutations were assessed. Partial viral compartmentalisation was present in nine of the ten participants. Broadly neutralising antibody (bnAb) escape was found to be associated with partial *env* compartmentalisation in some individuals, while cytotoxic T lymphocyte escape mutations in Gag were limited and did not differ between compartments. Viral compartmentalisation may be an important consideration for bnAb use in viral eradication.

## Highlights

• Partial *env/gag* compartmentalisation was present in nine of the ten participants.
• Cytotoxic T lymphocyte escape mutations (Gag) did not differ between compartments.
• Broadly neutralising antibody (bnAb) escape (Env) differed between compartments.
• Env compartmentalisation may be a consideration for bnAb use in viral eradication.

## 1 Introduction

Compartmentalisation of HIV variants has been reported in specific tissues including the central nervous system (CNS), gut associated lymphoid tissue as well as the male and female genital tracts (Schnell et al., 2010; Coombs et al., 1998; Miller et al., 2019; Overbaugh et al., 1996; Kemal et al., 2003; Bavaro et al., 2019; Nguyen et al., 2014; Stefic et al., 2017; van Marle et al., 2007; Wong et al., 1997; Sturdevant et al., 2012; Falcone et al., 2013). Variability of viruses present in different compartments may be an important consideration in the development of an effective preventative vaccine or cure for HIV (Stefic et al., 2017). For example, there could be signature mutations or characteristic features of variants in specific compartments that affect the viral sensitivity to cytotoxic T lymphocytes (CTLs) or broadly neutralising (bnAbs). Indeed, viral variants found in the cerebrospinal fluid (CSF) are less sensitive to bnAbs compared to variants found in the peripheral blood (Stefic et al., 2017), and signature differences in CTL epitopes between the spleen and CNS were reported (Wong et al., 1997).

There are conflicting reports for HIV compartmentalisation between lymph nodes and peripheral blood (Haddad et al., 2000; Van't Wout et al., 1998; Günthard et al., 2001). The lymph node is not only a major propagator of infection in untreated HIV infection, but during antiretroviral therapy (ART), the highest number of persistently infected cells are in lymphoid tissues (North et al., 2010; Kline et al., 2013). Therefore, it is important to understand whether the variants in lymph nodes have distinct genetic attributes compared to those in peripheral blood and to characterise these tissue-derived variants. However, most studies have focussed on peripheral blood.

In the present study, we investigated whether or not there is HIV-1 genetic compartmentalisation between the peripheral blood and lymph nodes through generating *env* and *gag* single genome amplicons (SGAs) from lymph nodes, plasma, and peripheral blood mononuclear cells derived from ten individuals infected with HIV-1 subtype C. We specifically assessed the *env* and *gag* genes to determine whether there are compartment-specific differences in bnAb escape mutations (in Env) and CTL escape mutations (in Gag), as such mutations have bearing on virus eradication approaches. Our results showed that partial compartmentalisation of both or either gene was present in most individuals, whether on ART or not. While there was an association between mutations affecting bnAb sensitivity and *env* compartmentalisation in some individuals, there was no association between CTL escape mutations in Gag and *gag* compartmentalisation.

## 2 Materials and methods

### 2.1 Study participants

Lymph nodes (LN), peripheral blood mononuclear cells (PBMC), and plasma (PL) samples were collected from individuals infected with HIV-1 subtype C from three cohorts in Durban, South Africa: the HIV Pathogenesis Programme acute infection cohort (Radebe et al., 2011), the “Females Rising through Education, Support and Health” (FRESH) cohort (Ndhlovu et al., 2015) and the Lymph Node cohort (Ogunshola et al., 2022). Study participants were chosen based on the availability of LN samples and the availability of PBMC and PL samples that were collected close to the LN excision time point. Of the ten individuals included in this study, six were untreated at the time of LN excision and the remaining four individuals were on ART at the time of LN excision (Table 1). PBMC and PL samples matched to the time of LN excision were studied in the untreated participants. In the treated participants, the matched PBMC and LN samples were compared to the PL sample collected immediately prior to ART initiation. The time point of each sample analysed is shown in Table 1. The study was approved by the Biomedical Research Ethics Committee of the University of KwaZulu-Natal (BREC/00002822/2021) and all participants provided written informed consent.

**Table 1:** Study participants and sample time points analysed.

PID	Gender	Age at LN excision	ART initiation^a^	PL viral Load^b^	PL_days^a^	LN^c^_days^a^	PBMC_days^a^	Genomic extractions	Gene analysed
**0011**	Female	26	Untreated	59000	1464	1464	1427	RNA (LN, PB, PL); DNA (LN, PB)	*gag* and *env*
**0053**	Female	26	Untreated	4100	921	926	921	RNA (PL); DNA (LN, PB)	*env*
**0108**	Female	24	Untreated	14000	50	55^d^	50	RNA (PL); DNA (LN, PB)	*gag* and *env*
**0118**	Male	29	Untreated	200000	0	Not done	0	RNA (PL); DNA (PB)	*gag* and *env*
**3011**	Female	20	Untreated	190000	0	3	0	RNA (PL); DNA (LN, PB)	*gag* and *env*
**079**	Female	25	Untreated	8000	1314	1315	1314	RNA (PL); DNA (LN, PB)	*gag* and *env*
**0098**	Female	26	270	300000	263	841	841	RNA (PL); DNA (LN, PB)	*gag* and *env*
**093**	Female	25	307	41000	249	1372^e^	1369	RNA (PL); DNA (LN, PB)	*gag* and *env*
**1012**	Female	25	0	6800	0	760	749	RNA (PL); DNA (LN, PB)	*gag* and *env*
**1210**	Female	22	0	110000	0	120	160	RNA (PL); DNA (LN, PB)	*gag*

PL (plasma), PBMC (peripheral blood mononuclear cells), LN (lymph node), PID (participant identifier), ART (antiretroviral therapy).

a Days post-enrollment.

b PL viral load is in copies/ml and corresponds to the time of PL collection. For treated participants, the PL sample was collected prior to ART initiation, while the PBMC and LN samples were collected after ART initiation. For untreated participants, the PL, PBMC and LN samples were collected at closely matched time points.

c Except where specified otherwise, the lymph node location was inguinal.

d Cervical location.

e Axillary location.

### 2.2 Sample processing and extractions

PL samples stored at −80 °C were thawed and RNA extraction was performed from 140 μl PL using the QIAamp Viral RNA Mini Kit (Qiagen, Hilden, Germany). For PL samples with viral loads less than 10 000 copies/ml, 500 μl of sample was concentrated by centrifugation at 14 000 RPM for 2 h prior to RNA extraction. LN and PBMC samples stored in liquid nitrogen were thawed and washed in phosphate buffered saline (ThermoFisher, Waltham, Massachusetts). DNA was extracted from 1 to 2 million LN cells using the MasterPure complete DNA and RNA extraction kit (Lucigen, Middleton, Wisconsin). DNA was extracted from 5 million PBMCs using the DNeasy blood and tissue kit (Qiagen). In addition to DNA, RNA was extracted from both LNs and PBMCs (1–2 million cells using the MasterPure complete DNA and RNA extraction kit) as the RNA is more likely to represent replicating variants.

### 2.3 Single genome amplification of HIV-1 env and gag

RNA was reverse transcribed into cDNA using the SuperScript IV Protocol (Invitrogen, Carlsbad, California) together with the gene-specific reverse primer. For the *env* amplification, the OFM19 primer was used while the Gag D_R primer was used for *gag* (all primer sequences available in Supplementary Table 1). SGAs of *env* and *gag* were generated using previously described methods (Gounder et al., 2017). To generate SGAs, nested PCR was implemented using endpoint dilutions of DNA or cDNA to obtain 30% PCR amplification (Salazar-Gonzalez et al., 2009). Briefly, first-round PCR was performed using the Platinum High Fidelity PCR System (Invitrogen) and primers OFM19 and VIF1. The second-round PCR was performed using the Phusion High Fidelity PCR System (ThermoFisher) and primers Env1A and Env1M. To generate *gag* SGAs, we performed a nested PCR using the Expand High Fidelity PCR System (Roche, Basel, Switzerland) with first round primers Gag D_F and Gag D_R, and second round primers Gag A_F and Gag C_R. All PCR products were viewed on a 1% agarose gel (ThermoFisher) containing gel red (ThermoFisher) to determine the presence of *env* and *gag* SGAs. A 1 kb GeneRuler (ThermoFisher) was loaded as a reference.

### 2.4 Sequencing

*Gag* and *env* sequencing was performed using the ABI Big Dye Terminator V3.1 cycle sequencing kit (Applied Biosystems, Foster City, California) and the sequencing primers listed in Supplementary Table 1. Sequences were generated using the ABI 3130xl Genetic Analyser. The Sequencher software program version 5.4.6 (Gene Codes Corporation, Ann Arbor, Michigan) was used to assemble and manually edit the overlapping DNA fragments. Edited sequences were then aligned, and maximum likelihood phylogenetic trees were also constructed using PHYML for each compartment and participant using the Geneious software v10.1.3 (Biomatters Ltd, Auckland, New Zealand). Hypermutants were identified by using the Hypermut 2.0 tool available at www.hiv.lanl.gov (Fisher's exact p < 0.05). Sequences are available under GenBank accession numbers ON552408-ON552416 and OQ554543-OQ554954.

### 2.5 Compartmentalisation analysis

Hyphy analysis was performed using the Hyphy software (Pond et al., 2005). This analysis included Wright's measure of population subdivision (F-statistic, FST), tree-based Slatkin-Madison (SM) test, nearest neighbour statistic test (Snn), Simmonds association index (AI) and correlation coefficients (r, rb) (Pond et al., 2005). The presence or absence of compartmentalisation as well as the degree of compartmentalisation (partial or complete) was determined according to the p values from the SM and FST test, and the phylogenetic tree appearance, based on previous studies showing phylogenetic trees with varying degrees of compartmentalisation (Schnell et al., 2010, 2011). Briefly, complete compartmentalisation was defined as SM and FST p values of ≤0.0001 as well as near complete separation of compartments in a phylogenetic tree; partial compartmentalisation was defined as SM and FST p values of ≤0.05 and > 0.0001 as well as incomplete separation of compartments in a phylogenetic tree; and equilibration was defined as SM and FST p values of >0.05 and no separation of compartments in a phylogenetic tree.

### 2.6 Analysis of CTL escape mutations within HIV-1 Gag

To investigate whether compartmentalisation was associated with compartment-specific differences in CTL escape mutations within *gag*, previously documented CTL escape mutations were identified in each sequence. To do this, a list of CTL variants in Gag was downloaded from the HIV Los Alamos immunology database (http://www.hiv.lanl.gov/, accessed 5 November 2021). This list was filtered to include only experimentally confirmed CTL escape mutations, which included the following terms in the database: diminished HLA binding or increased off-rate, diminished response, escape documented, non-susceptible form, TCR related mutation, literature escape, and processing. The total number of mutations present were then documented for each Gag sequence. In addition, it was documented whether these mutations were in epitopes restricted by the participant HLA alleles, as those mutations in participant HLA-restricted epitopes could possibly have been selected in the patient.

### 2.7 Analysis of bnAb escape mutations within HIV-1 Env

To determine whether bnAb escape mutations contributed to *env* compartmentalisation, a list of bnAb escape mutations, or mutations that affect neutralisation sensitivity, was compiled from previously published work. The list of mutations was derived from the following *env* regions: the CD4 binding site (Caskey et al., 2017; Schommers et al., 2020; Huang et al., 2016; Lynch et al., 2012, 2015; Crooks et al., 2018; Wibmer et al., 2015; Bar et al., 2016; Otsuka et al., 2018; Li et al., 2011), the V1V2 region (Schommers et al., 2020; Crooks et al., 2018; Wibmer et al., 2015; van der Velden et al., 2020; Lee et al., 2017; Moore et al., 2013), the V3 region (Caskey et al., 2017; Wibmer et al., 2015; Krumm et al., 2016; Murphy et al., 2013; Bricault et al., 2019; Chan et al., 2021; Anthony et al., 2017; Moore et al., 2009), the gp120/40 region (Huang et al., 2016; Wibmer et al., 2015) and the MPER region (Wibmer et al., 2015; Gray et al., 2008). These mutation sites were compared across compartments - sequences were aligned with HXB2 (accession number: K03455) and the South African consensus C sequence (http://www.hiv.lanl.gov/) to assign codon position numbers and identify which amino acids were consensus or non-consensus.

## 3 Results

### 3.1 Phylogenetic analysis of *gag* and *env* SGAs

The aim of this study was to generate approximately ten SGA sequences for both *env* and *gag* genes in each compartment (PBMC DNA, PBMC RNA, LN DNA, LN RNA and PL RNA) for ten participants to use in compartmentalisation analyses. A total of 171 *env* SGA sequences (7–12 per compartment) were successfully generated from five of the six untreated participants and one of the four treated participants (Table 2). A total of 250 *gag* sequences (7–11 per compartment) were generated from five of the six untreated participants and all four treated participants (Table 2). A full complement of sequences for all compartments was not obtained due to limitations in sample availability and/or technical difficulties in HIV amplification, particularly from PBMC RNA and LN RNA. PBMC RNA and LN RNA sequences were only generated for participant 0011. Therefore, for all other participants, compartmentalisation analysis was based on PL, PBMC DNA and LN DNA only.

**Table 2:** Total *env* and *gag* single genome amplicons generated.

PID	Gene	PBMC DNA	PBMC RNA	LN DNA	LN RNA	PL
**0011**	*env*	10	10 (1)^a^	10 (1)^a^	5	8
	*gag*	10 (#1)^a^	10	11	2	9
**0053**	*env*	10		10		9
**0108**	*gag*	9		9		8
**079**	*env*	12		10 (#1)^a^		7
	*gag*	10		10		10
**118**	*env*	9 (1)^a^				8
	*gag*	8				8
**3011**	*env*	10		8 (#2)^a^		9
	*gag*	10		9		8
**093**	*env*	9 (#2)^a^		7		10
	*gag*	9 (#1)^a^		9 (#1)^a^		11
**0098**	*gag*	9		9		7
**1012**	*gag*	9		11 (1, #3)^a^		8
**1210**	*gag*	7		11		9

PL (plasma), PBMC (peripheral blood mononuclear cells), LN (lymph node), PID (participant identifier).

a The number in brackets refers to the number of defective sequences with internal stop codons (these form part of the total number listed in the table). Those with a # are hypermutants as identified by using the Hypermut 2.0 tool available at www.hiv.lanl.gov (Fisher’s exact p < 0.05).

**Fig. 1:**
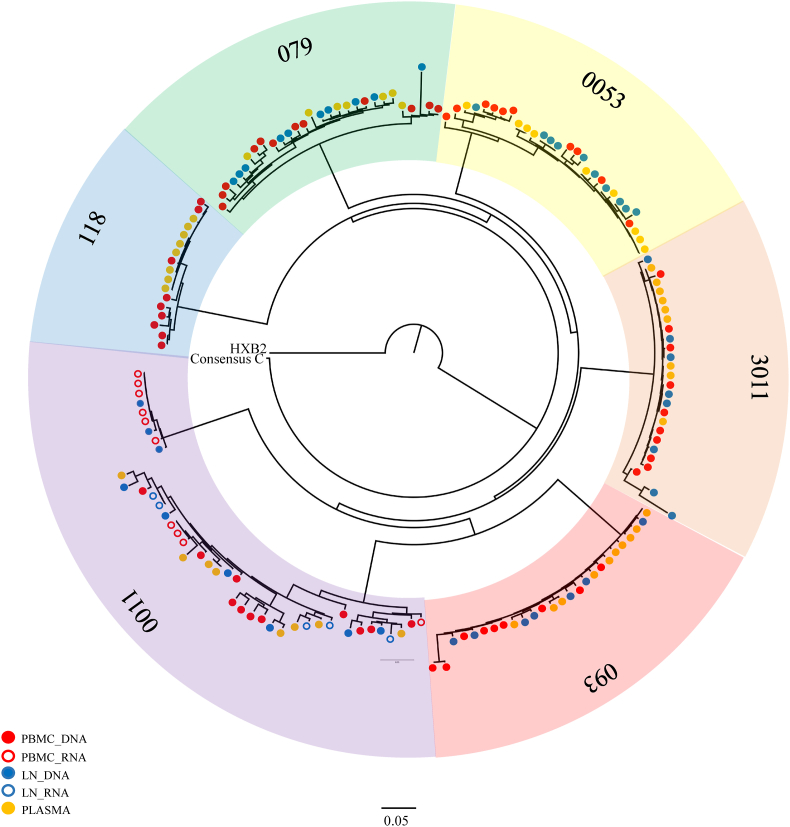
**Phylogenetic tree of HIV-1 *env* single genome sequences.** Sequences for each participant are shaded in a different colour. Participant 093 is treated, while all other participants are untreated. The plasma, peripheral blood mononuclear cells (PBMC) and lymph node (LN) compartments from which sequences were generated are represented by open or closed circles of different colours as shown in the key. Reference sequences included were HXB2 (accession no. K03455) and consensus C (http://www.hiv.lanl.gov/). The tree was generated using PhyML on Geneious Version 1.3.2 (Biomatters).

**Fig. 2:**
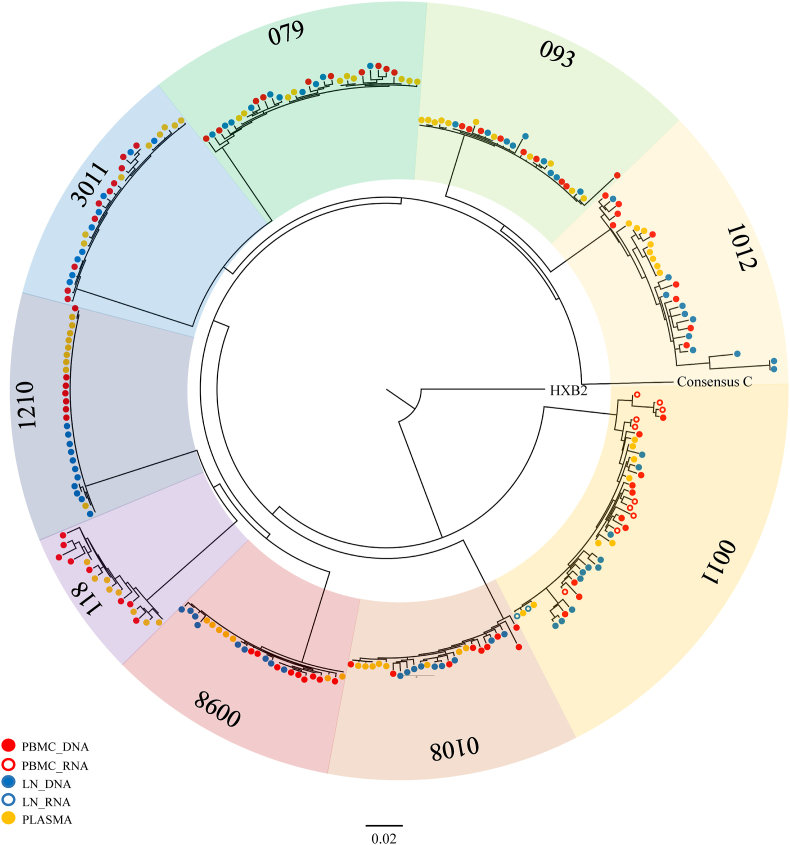
**Phylogenetic tree of HIV-1 *gag* single genome sequences.** Sequences for each participant are shaded in a different colour. Participants 093, 0098,1012 and 1210 are treated, while participants 0011, 0108, 079, 3011 and 118 are untreated. The plasma, peripheral blood mononuclear cells (PBMC) and lymph node (LN) compartments from which sequences were generated are represented by open or closed circles of different colours as shown in the key. Reference sequences included were HXB2 (accession no. K03455) and consensus C (http://www.hiv.lanl.gov/). The tree was generated using PhyML on Geneious Version 1.3.2 (Biomatters).

Phylogenetic trees for both *env* (Fig. 1) and *gag* (Fig. 2) were constructed to assess the relatedness of sequences. For both *env* and *gag,* sequences from the same patient clustered together and all sequences clustered with the consensus C reference sequence. With few exceptions, sequences from untreated participants showed greater diversity than those from treated participants. Interestingly, untreated participant 0011 had notably higher sequence diversity than other participants as well as 2 distinct clusters of sequences. Contamination was ruled out as the cause of the 2 distinct 0011 clusters by performing different extractions, amplifications and sequencing runs at different times.

### 3.2 Compartmentalisation analysis

**Fig. 3:**
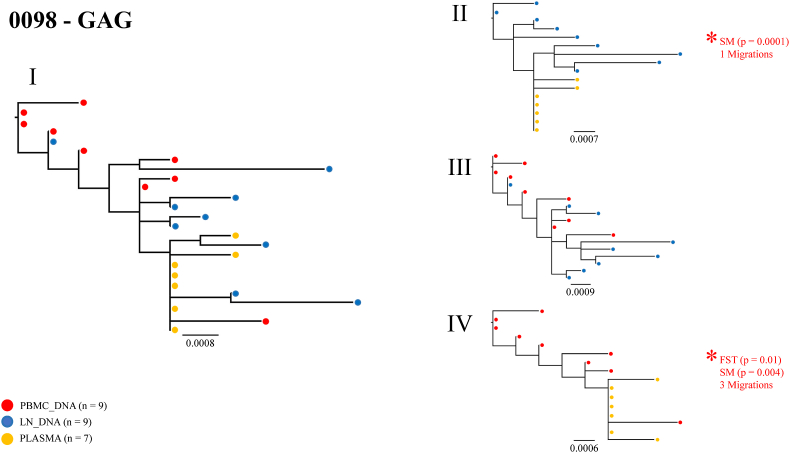
**Phylogenetic trees and compartmentalisation analysis of HIV-1 *gag* sequences for participant 0098.** Sequences for participant 0098 were phylogenetically analysed and used to perform statistical analysis (Hyphy) to determine the presence of compartmentalisation. Phylogenetic trees were constructed from *gag* single genome sequences derived from different compartments. I) Phylogenetic tree showing relatedness of sequences derived from all compartments, II) LN_DNA and plasma, III) PBMC_DNA and LN_DNA, and IV) PBMC_DNA and plasma. Red circles represent PBMC_DNA, blue circles represent LN_DNA, and yellow circles represent plasma. The number of SGAs generated per compartment are shown in brackets in the key. Statistically significant results (significant F-statistic [FST] and/or Slatkin-Madison [SM] tests) are shown with an asterisk (*) with accompanying p-values in red. Panel II shows complete compartmentalisation, panel III shows equilibration and panel IV shows partial compartmentalisation.

To investigate whether there was evidence of *env* and/or *gag* compartmentalisation, a compartment-by-compartment analysis was performed for each participant, where two compartments were compared at a time. For each two-way comparison, complete compartmentalisation, partial compartmentalisation, or equilibration was assigned, which was based on statistical results from the HyPhy analysis as well as the intermingling or clustering observed in phylogenetic trees. A detailed analysis of one participant (0098) who showed all the varying degrees of compartmentalisation across the different two-way comparisons of the *gag* gene is shown in Fig. 3, while the detailed analysis of the remaining participants is in Supplementary Figs. 1–9.

The compartmentalisation results for each gene are summarised in Table 3. Overall, partial compartmentalisation was present in nine of ten participants, where it was either present in both genes, only in *env*, or only in *gag*, and not consistently between the same compartments. There was no consistent link between the presence or absence of compartmentalisation and treatment status. When comparing the LN DNA and PL compartments, a gene-specific pattern was observed - partial compartmentalisation was observed in *gag* for six of nine participants, while there was partial compartmentalisation in *env* for only two of five participants. Similarly, there was a gene-specific pattern when comparing PBMC DNA and PL – partial compartmentalisation in *env* was observed for five of six participants and in *gag* for only two of nine participants. Interestingly, in the majority of participants there was equilibration for both genes between LN DNA and PBMC DNA compartments. In summary, there were gene-specific patterns in which particular compartments showed partial *env/gag* compartmentalisation.

**Table 3:** Summary of HIV-1 *gag/env* compartmentalisation results.

PID	Treatment	Gene^a^	Compartment-by-compartment analysis					
			PBMC RNA PBMC DNA	PBMC RNA LN DNA	PBMC RNA PL	PBMC DNA LN DNA	PBMC DNA PL	LN DNA PL
**0011**	No	*env*	Partial	Equilibrated	Equilibrated	Equilibrated	Partial	Equilibrated
		*gag*	Equilibrated	Partial	Equilibrated	Equilibrated	Equilibrated	Partial
**0053**	No	*env*	–	–	–	Partial	Partial	Equilibrated
**0108**	No	*gag*	–	–	–	Equilibrated	Equilibrated	Partial
**079**	No	*env*	–	–	–	Equilibrated	Equilibrated	Equilibrated
		*gag*	–	–	–	Equilibrated	Partial	Partial
**118**	No	*env*	–	–	–	–	Partial	–
		*gag*	–	–	–	–	Equilibrated	–
**3011**	No	*env*	–	–	–	Equilibrated	Partial	Partial
		*gag*	–	–	–	Equilibrated	Equilibrated	Partial
**093**	Yes	*env*	–	–	–	Equilibrated	Partial	Partial
		*gag*	–	–	–	Equilibrated	Equilibrated	Equilibrated
**0098**	Yes	*gag*	–		–	Complete	Equilibrated	Partial
**1012**	Yes	*gag*	–	–	–	Equilibrated	Partial	Partial
**1210**	Yes	*gag*	–	–	–	Equilibrated	Equilibrated	Equilibrated

PL (plasma), PBMC (peripheral blood mononuclear cells), LN (lymph node), PID (participant identifier).

a Rows with *gag* compartmentalisation results are shaded, while rows with *env* compartmentalisation results are not shaded.

### 3.3 Association between CTL escape and compartmentalisation

**Fig. 4:**
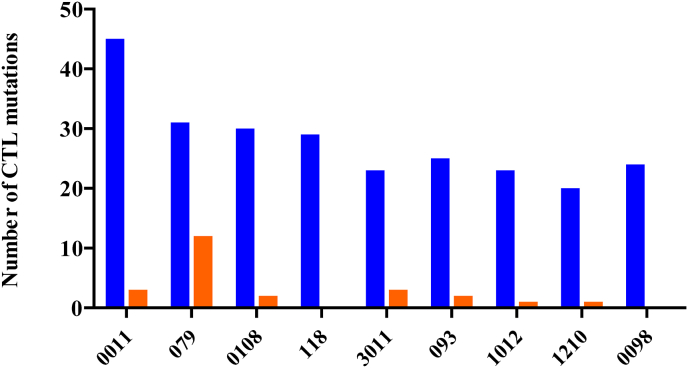
**CTL escape mutations in Gag.** Experimentally confirmed cytotoxic T lymphocyte (CTL) escape mutations (http://www.hiv.lanl.gov/) in Gag were quantified for each participant. A) The total number of CTL mutations that were present for each participant is represented in blue, while the total number of CTL mutations in epitopes restricted by the participant human leukocyte antigen (HLA) class I alleles is represented in orange. There was no HLA data collected for participants 118 and 0098.

To explore whether CTL escape mutations may contribute to *gag* compartmentalisation, the number of these mutations present were documented for each sequence and compared between compartments. There were 20–45 CTL escape mutations present in each participant, where the minority were in epitopes restricted by the patient HLA alleles (Fig. 4). Table 4 shows CTL escape mutations that were ≥30% different in frequency between compartments. With the exception of participant 0011, most participants had 0-2 CTL escape mutations that differed in frequency between compartments by ≥ 30%, and this was not linked to compartmentalisation patterns for *gag.* In summary, there was no association between CTL escape and *gag* compartmentalisation.

**Table 4:** Frequency (%) of CTL escape mutations differing by ≥ 30% between compartments.

PID	Mutation	LN DNA	LN RNA	PBMC DNA	PBMC RNA	PL
**0011**	K12 N^a^	100	100	90	70	100
	K28Q	0	0	30	30	0
	Q28K^b^	100	100	60	50	100
	H28S	0	0	10	20	0
	K28Q, K30R	0	0	30	30	0
	P28S	0	0	10	20	0
	K30R, I34L	0	0	10	20	0
	L215 M^b^	64	100	80	50	78
	I247V, G248A	0	0	10	20	0
	V247I	100	100	90	80	89
	V247I, G248A	100	100	90	80	89
	N252S, I256V^b^	82	100	40	20	78
	S252G, I256V^b^	82	100	40	20	78
	A374T	73	100	90	100	100
**079**	T310S	40		70		80
	S310T, E312D^b^	70		30		20
**118**	A224P			63		25
**3011**	K28Q^a^	89		70		100
	K28R^a^	11		30		0
**093**	R403K	67		44		18
**1012**	S332T	36		33		0

PL (plasma), PBMC (peripheral blood mononuclear cells), LN (lymph node), PID (participant identifier), CTL (cytotoxic T lymphocyte).

a Mutations in epitopes restricted by participant-specific HLA alleles.

b Mutations differing by ≥ 50% between compartments.

### 3.4 Association between bnAb escape mutations and compartmentalisation

Mutations at sites previously reported to affect sensitivity to bnAbs were documented for each sequence and compared between compartments, to explore whether bnAb escape may contribute to *env* compartmentalisation. All participants had mutations (whether fixed, dominant or at a low frequency) at sites reported to affect bnAb sensitivity, and all but one participant (079) had mutations in all 5 antibody-binding regions - the CD4 binding site, V1V2, V3, gp120/41 and MPER regions (Supplementary Figs. 10–14). Participant 0011 had the highest number of mutations present in each region. Participant 079 was fairly conserved throughout all 5 regions, and completely conserved in the gp120/41 region.

The frequency of mutations was compared between compartments and those that differed between compartments by ≥ 50% are shown in Table 5 and highlighted in Supplementary Figs. 10–14. Participants 0011, 0053 and 118 were the only participants for whom compartment-specific variability of ≥50% was present, and this was related to *env* compartmentalisation results for these participants. In participant 0011, the differing frequency of mutations between PBMC DNA and PBMC RNA compartments may have contributed to the partial *env* compartmentalisation between PBMC DNA and PBMC RNA (Table 3). Based on previously reported effects of different amino acid variants at these bnAb sites (Table 5), these mutation frequency differences are expected to translate into a higher frequency of bnAb resistance in the PBMC RNA compartment compared to the PBMC DNA compartment in participant 0011. In participant 0053, the PBMC compartment had mutations that differed in frequency by 50% or more when compared to the LN and PL compartments, which was coincident with partial *env* compartmentalisation between PBMC and LN/PL in this patient (Table 3). Based on previously reported effects of different amino acid variants at bnAb sites in the CD4 binding region, V1V2 region, gp120/41 regions (Table 5), the mutation frequency differences observed in participant 0053 are expected to translate into a lower frequency of bnAb resistance in the PBMC compartment. Similarly, participant 118 showed both partial *env* compartmentalisation between PBMC DNA and PL compartments (Table 3) and multiple mutations differing by ≥ 50% between PBMC DNA and PL compartments, where most of these differences were consistent with a higher frequency of bnAb resistance in PL compared to the PBMC compartment (based on literature (Table 5)). In summary, bnAb escape could partly contribute to the *env* compartmentalisation observed in participants 0011, 0053 and 118.

**Table 5:** Frequency (%) of bnAb escape mutations differing by ≥ 50% between compartments.

PID	Region	AA mutation		Con C	LN DNA	LN RNA	PBMC DNA	PBMC RNA	PL	Effect in literature	Ref
**0011**	CD4 binding site	234	D	N	30	0	0	60	0	Removal of 234 glycan confers resistance to 8ANC195	West et al. (2013)
		281	I	A	30	0	0	60	0	VRC01 escape due to mutations between positions 278 and 281	Lynch et al. (2015)
		282	N	K	30	0	0	70	0	K282A showed resistance to 3BNC117, VRC-PG04 and 12A21	Huang et al. (2016)
		371	V	I	30	0	0	60	0	Removal of I371 results in reduced binding of b2 and VRC01	Lynch et al. (2012)
		455	V	T	70	100	100	40	100	455T escapes antibody 3BNC117	Caskey et al. (2015)
		474	N	D	60	20	20	90	38	D474 mutation confers diminished response to VRC01 neutralisation	Li et al. (2011)
	V1V2	160	T	N	30	0	0	60	0	PG9/16 antibody dependant on N160 glycan.	Wibmer et al. (2015)
		162	A	T	30	0	0	60	0	T162I/N resistant to PG16 and PGDM1400	Schommers et al. (2020)
		166	K	R	30	0	0	60	0	CAP256 BCN antibody dependant on R166 for neutralisation	Moore et al. (2011)
		167	G	D	20	20	0	50	0	CAP256 BCN antibody dependant on D167 for neutralisation	Moore et al. (2011)
		171	E	K	30	0	0	60	0	CAP256 BCN antibody dependant on K171 for neutralisation	Moore et al. (2011)
		171	Q	K	70	100	60	40	87.2	CAP256 BCN antibody dependant on K171 for neutralisation	Moore et al. (2011)
	V3	328	K	Q	40	40	70	40	75	Within the recognition site for antibodies targeting the V3 region	Wibmer et al. (2015)
		339	D	N	70	100	100	40	10	I339 N confers escape from the anti C3 antibody in CAP88	Moore et al. (2009)
	gp120/41	230	D	N	30	0	0	60	0	35O22 and PGT151 dependant on glycan present at 230 for neutralisation	Wibmer et al. (2015)
		234	D	N	30	0	0	60	0	8ANC195 dependant on glycan present at 234 for neutralisation	Wibmer et al. (2015)
		448	T	N	70	100	100	40	100	Variants lacking N448 escaped bnAb SF5/SF12	Schoofs et al. (2019)
	MPER	671	S	N	70	100	100	40	100	In the target site for 4E10 and 2F5	Gray et al. (2008)
		674	S	D	60	20	20	70	13	D674S, D674E and D674T mutations are resistant to neutralisation by PGZL1, H4K3, 10E8 and 4E10	Zhang et al. (2019)
		676	S	T	30	40	50	0	38	In the target site for 4E10 and 2F5	Gray et al. (2008)
		683	N	K	30	0	0	60	0	In the target site for 4E10 and 2F5	Gray et al. (2008)
		683	T	K	70	100	100	40	100	In the target site for 4E10 and 2F5	Gray et al. (2008)
**0053**	CD4 binding site	279	D	N	90	–	40	–	83	N279K and N279E confers resistance to VRC01 bnAbs	Lynch et al. (2015)
		463	S	T	90	–	40	–	89	463D, R, E escapes antibody BNC117	Caskey et al. (2015)
	V1V2	171	R	K	50	–	0	–	44	CAP256 BCN antibody dependant on K171 for neutralisation	Moore et al. (2011)
	gp120/41	279	D	N	90	–	40	–	83	N279K and N279E confers resistance to VRCO1 bnAbs	Lynch et al. (2015)
	MPER	677	E	N	40	–	90	–	100	N677K increased the sensitivity of bnAb 4E10 by threefold. Mutations at this position may affect presentation of the 4E10 epitope	Gray et al. (2008)
		677	K	N	60	–	10	–	0	N677K increased the sensitivity of bnAb 4E10 by threefold. Mutations at this position may affect presentation of the 4E10 epitope	Gray et al. (2008)
		683	R	K	40	–	90	–	89	In the target site for 4E10 and 2F5.	Gray et al. (2008)
**118**	CD4 binding site	276	D	N	–	–	63	–	0	Removal of the glycan confers resistance to HJ16. Increases sensitivity to VRC01 and VRC03	Balla-Jhagjhoorsingh et al. (2013)
	V1V2	169	N	K	–	–	33	–	88	CAP256 BCN antibody dependant on K169 for neutralisation	Moore et al. (2011)
		169	R	K	–	–	50	–	0	CAP256 BCN antibody dependant on K169 for neutralisation	Moore et al. (2011)
	V3	334	N	D	–	–	33	–	100	Mutations at site 334 produces escape against 10-1074	Caskey et al. (2017)
	gp120/41	276	D	N	–	–	0	–	63	Removal of 276 glycan increases resistance to 8ANC195	West et al. (2013)
	MPER	674	N	D	–	–	33	–	100	D674S, D674E and D674T mutations are resistant to neutralisation by PGZL1, H4K3, 10E8 and 4E10	Zhang et al. (2019)
		683	Q	K	–	–	33	–	100	In the target site for 4E10 and 2F5	Gray et al. (2008)

PL (plasma), PBMC (peripheral blood mononuclear cells), LN (lymph node), PID (participant identifier), bnAbs (broadly neutralising antibodies), Ref (references).

## 4 Discussion

In this study we aimed to investigate whether there is viral compartmentalisation between LN, PBMC and PL compartments. The *env* and *gag* genes were analysed to determine whether or not compartmentalisation is gene-specific and if there are compartment-specific differences in bnAb escape mutations and CTL escape mutations, as such mutations have bearing on virus eradication approaches. Partial virus compartmentalisation between at least two compartments was present in most individuals (nine of ten participants), and gene-specific patterns of compartmentalisation were observed. Lastly, an association between *env* compartmentalisation and the presence of bnAb escape mutations was observed in some participants, yet no association between CTL escape and *gag* compartmentalisation was observed.

Compartmentalisation occurred mostly between PBMC and PL or LN and PL. In treated participants, it is not known whether compartmentalisation between LN/PBMC and PL was already present at the time of therapy initiation since PL samples were collected prior to therapy initiation while LN and PBMC samples were collected after therapy initiation. Results from untreated participants, where all six showed partial compartmentalisation between LN/PBMC and PL and all compartments were sampled at a similar time point, indicate that the presence of compartmentalisation at the time of therapy initiation is a strong likelihood. It is also possible that compartmentalisation between PBMC/LN and PL in treated participants may have developed in part after the PL samples were collected, due to possible low levels of viral replication during the time to viral suppression (Fletcher et al., 2014a) or clonal expansion of proviral DNA during treatment (Schnell et al., 2010). Low-level replication during treatment in LN tissue is a possibility when considering that drug penetration may be less effective in this compartment, although this remains controversial (Fletcher et al., 2014b).

The presence of viral compartmentalisation between PBMC and PL was less expected than that between LN and PL since PBMC and PL are both derived from the peripheral blood. The presence of compartmentalisation between PBMC and PL was attributed to multiple possibilities. Firstly, there could be variability in the source of virus infecting PBMCs and PL (Simmonds et al., 1991). In addition, PBMCs are self-sustaining in their viral infection (additional variation can develop within PBMCs). Further, a portion of the integrated proviruses in PBMCs may be defective, while the PL virus represents replication-competent virus only (Simmonds et al., 1991). The presence of non-integrated linear or circular DNA, which is abundant in PBMCs and could be going through recombination, may be an added factor contributing to compartmentalisation between PBMCs and PL (Hamid et al., 2017; Burgard et al., 2000).

Compartmentalisation was observed between PBMC RNA and PL in one participant for whom PBMC RNA could be generated. In this participant (0011), there was a group of sequences (predominantly from the PBMC RNA compartment) that clustered separately from the main 0011 cluster in the phylogenetic tree, while remaining separate from other patient clusters (Fig. 1). A possible explanation is that this participant had multiple transmission events, however, longitudinal sampling would be required to test this possibility. Alternatively, the small separate cluster could represent defective sequences, or non-translating sequences (intracellular RNA is made up of both translating RNA and non-translating RNA (Chen et al., 2020)), especially since PL sequences are not represented in this cluster. Infectivity experiments on the *env* SGAs from this small cluster would be required in order to address this hypothesis.

Virus compartmentalisation was not observed between LN and PBMC compartments in most (all four treated and five of six untreated) individuals. This is in line with several previous studies done on treated participants, which analysed either the p6-RT region, the *env* gene or full length sequences (van't Wout et al., 1998; McManus et al., 2019; Kuo et al., 2020; Bozzi et al., 2019), as well as a study in untreated participants showed equilibration of *env* V3 and *pol* sequences between the LN and PBMC compartments (Haddad et al., 2000). While those studies, together with the present analysis, suggest that there is no restriction in gene flow between the LN and PBMC compartments in most cases, there was a study that reported viral compartmentalisation (in *env* and *pol*) between LN and PBMC compartments in treated participants who had developed drug resistance (Haddad et al., 2000). It was suggested that this was likely due to the unequal distribution of antiretroviral drugs between LN and PBMC compartments.

A different compartmentalisation pattern between *env* and *gag* genes was observed. Most participants showed partial *env* compartmentalisation between PBMC and PL and partial *gag* compartmentalisation between LN and PL. The mechanism underlying this observation is unclear. Cell types, immune responses and even co-infections that alter the micro-environment might differ between the compartments and it is plausible that these could apply different pressures on different parts of the virus (Blackard, 2012), however it is then surprising that compartmentalisation directly between the LN and PBMC in either *gag* or *env* was rarely present.

In this study, an association between *env* compartmentalisation and bnAb escape was observed. In a previous study, which analysed bnAb resistance in association with compartmentalisation, bnAb resistant variants were present in the CNS but not in the peripheral blood (Stefic et al., 2017). In the present study, in a few participants there were multiple mutations at *env* sites that are reported to affect sensitivity to bnAbs, where these mutations were at least 50% different in frequency between compartments and were coincident with partial *env* compartmentalisation between the same compartments. These mutations included those that confer resistance to bnAbs that are currently in clinical trials, such as VRC01 and 10–1074 (Spencer et al., 2021; Grobben et al., 2019). Further characterisation of bnAb escape mutation differences between compartments, including neutralisation sensitivity assays to confirm the effect on bnAb sensitivity, will better inform bnAb eradication approaches. It would also be of interest to conduct a longitudinal analysis to track how the bnAb mutations arise and whether fitness costs of mutations as well as ease of compensation could be related to compartmentalisation.

The few CTL escape mutations in Gag that differed in frequency between compartments did not coincide with compartmentalisation. Consistent with these results, previous studies have shown that CTL escape is similar between different anatomical locations where the virus is compartmentalised (Miller et al., 2019; Kelentse et al., 2020). It is possible that the immune environment is not sufficiently different between the compartments studied to drive significant differences in Gag CTL escape mutations between compartments, and the conserved nature of Gag when compared to Env may also be partly responsible. These results suggest that a Gag specific CTL-based vaccine is likely to have similar effectiveness across the different compartments studied here.

One limitation of the current analysis was the cross-sectional nature as well as the varying stages of infection in the participants. Studying a group of participants with similar clinical characteristics longitudinally and synchronising time points, may allow a better picture of how and when HIV-1 compartmentalisation occurs. Additionally, difficulty was experienced in amplifying *env* and *gag*, either due to sample amount limitations or technical challenges in amplifying from extracted viral RNA. Therefore, a limited number of SGAs were studied. However, the optimum number of SGAs per compartment, to give an accurate representation of compartmentalisation, is between 20 and 30 SGAs (Zárate et al., 2007). Another limitation of the current analysis is that the compartmentalised sequences were not interrogated using functional assays to confirm that they represented functional sequences and it is also unknown whether they are present within a fully functional genome. It should also be noted that the PBMC sample from participant 0011 was collected a month apart from the PL and LN samples, and this could have had the potential to affect compartmentalisation results for this participant. Nevertheless, the PBMC DNA conformed to the general patterns observed – PBMC DNA was equilibrated with LN DNA for both genes and there was partial *env* (but not *gag*) compartmentalisation between PBMC DNA and PL.

## 5 Conclusion

In summary, the results show that *env/gag* compartmentalisation is present in most HIV-infected individuals, and that *env* compartmentalisation is partly associated with mutations reported to alter bnAb sensitivity. Further study to characterise bnAb escape in different compartments is warranted.

## CRediT authorship contribution statement

**Neschika Jeewanraj:** Investigation, Formal analysis, writing. **Tawanda Mandizvo:** Investigation. **Takalani Mulaudzi:** Investigation. **Nombali Gumede:** Investigation. **Zaza Ndhlovu:** Resources, Funding acquisition. **Thumbi Ndung'u:** Resources, Conceptualization, Funding acquisition. **Kamini Gounder:** Conceptualization, Supervision, writing. **Jaclyn Mann:** Conceptualization, Funding acquisition, Supervision, writing, All authors reviewed the final draft.
