# Corrigendum to “Partial compartmentalisation of HIV-1 subtype C between lymph nodes, peripheral blood mononuclear cells and plasma” [Virology 582 (2023) 62–70]

**DOI:** 10.1016/j.virol.2023.05.010

**Published:** 2023-08

**Authors:** Neschika Jeewanraj, Tawanda Mandizvo, Takalani Mulaudzi, Nombali Gumede, Zaza Ndhlovu, Thumbi Ndung'u, Kamini Gounder, Jaclyn Mann

**Affiliations:** a HIV Pathogenesis Programme, Doris Duke Medical Research Institute, Nelson R. Mandela School of Medicine, University of KwaZulu-Natal, Durban, South Africa; b Africa Health Research Institute, Durban, South Africa; c Ragon Institute of Massachusetts General Hospital, Massachusetts Institute of Technology and Harvard University, Cambridge, MA, USA; d Division of Infection and Immunity, University College London, London, United Kingdom

The authors regret omission of acknowledgement of a funder, and incorrect entries on two lines of Table 3:

1. This work was also supported in part by a grant from the Harvard University Center for AIDS Research (CFAR), an NIH-funded program (P30 AI060354).
2. Corrections to Table 3:

**Table 3:**
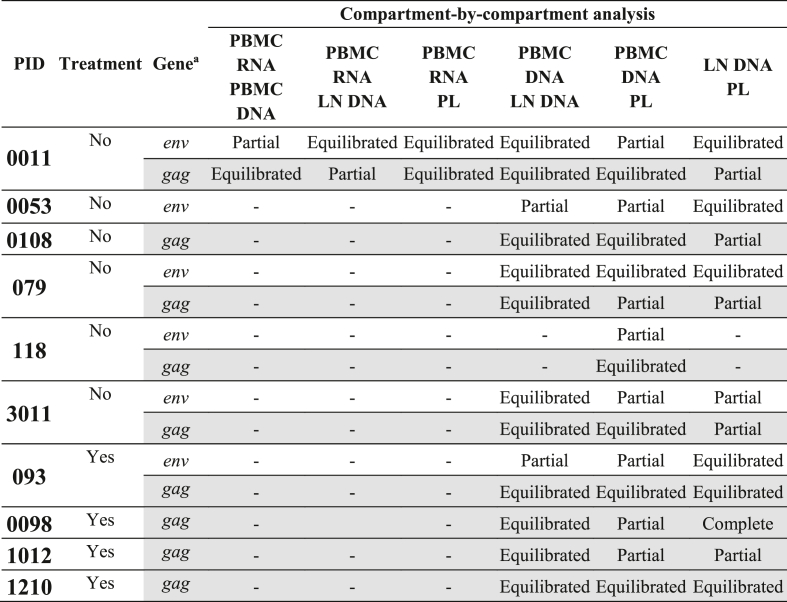
Summary of HIV-1 *gag/env* compartmentalisation results.

PL (plasma), PBMC (peripheral blood mononuclear cells), LN (lymph node), PID (participant identifier).

^a^ Rows with *gag* compartmentalisation results are shaded, while rows with *env* compartmentalisation results are not shaded.

**Correction to text associated with Table 3:**

When comparing the LN DNA and PL compartments, a gene-specific pattern was observed - partial compartmentalisation was observed in *gag* for six of eight participants, while there was partial compartmentalisation in *env* for only one of five participants. Similarly, there was a gene-specific pattern when comparing PBMC DNA and PL – partial compartmentalisation in *env* was observed for five of six participants and in *gag* for only three of nine participants.

The authors would like to apologise for any inconvenience caused.

