# Supplementary material for: Partial compartmentalisation of HIV-1 subtype C between lymph nodes, peripheral blood mononuclear cells and plasma
Source: Virology. 2023 May;582:62–70. doi: 10.1016/j.virol.2023.03.011 (PMC10132742; doi:10.1016/j.virol.2023.03.011)
Supplement: Multimedia component 1 [file mmc1.docx]

**Supplementary Table 1. List of *env* and *gag* primers used for nested PCR and sequencing**

| **Primer name** | **Primer** | **Method** | **Gene** | **HXB2 position** |
| --- | --- | --- | --- | --- |
| **OFM19** | 5′ - GCA CTC AAG GCA AGC TTT ATT GAG GCT TA-3′ | PCR (SGA) | *env* | 9604-9632 |
| **VIF1** | 5′ - GGG TTT ATT ACA GGG ACA GCA GA -3′ | PCR (SGA) | *env* | 4900-4923 |
| **Env1A** | 5′ - CAC CGG CTT AGG CAT CTC CTA TGG CAG GAA GAA-3′ | PCR (SGA) | *env* | 5954–9096 |
| **Env1M** | 5′ - TAG CCC TTC CAG TCC CCC CTT TTC TTT TA -3 | PCR (SGA) | *env* | 9096–9068 |
| **sq6rc(2)** | 5’ GAATTGGGTCAAAAGAGACCTTTGGA 3’ | Sequencing | *env* | 6839-6864 |
| **ef00** | 5’ AAA GAG CAG AAG ACA GTG GCA ATG A 3’ | Sequencing | *env* | 6204-6228 |
| **sq5.5rc** | 5’ CTAGGAGCTGTTGATCCTTTAGGTAT 3’ | Sequencing | *env* | 7979-8004 |
| **sq13f(2)c** | 5’ TATATAAATATAAAGTGGTAGAAATTAAGC 3’ | Sequencing | *env* | 7672-7701 |
| **sq14fc** | 5’ ACTCACGGTCTGGGGCATTA 3’ | Sequencing | *env* | 7925-7944 |
| **sq3r(2)c** | 5’ GCTATGGTATCAAGCAGACTAATAGCACTC 3’ | Sequencing | *env* | 8651-8680 |
| **Gag A_F** | 5’- CTC TCG ACG CAG GAC TCG GCT T - 3’ | PCR (SGA) and Sequencing | *gag* | 683–704 |
| **Gag A_R** | 5’- ACA TGG GTA TCA CTT CTG GGC T - 3’ | Sequencing | *gag* | 1282–1303 |
| **Gag B_F** | 5’- CCA TAT CAC CTA GAA CTT TGA AT - 3’ | Sequencing | *gag* | 1226–1246 |
| **Gag B_R** | 5’- CTC CCT GAC ATG CTG TCA T - 3’ | Sequencing | *gag* | 1825–1846 |
| **Gag C_F** | 5’- CCT TGT TGG TCC AAA ATG CGA - 3’ | Sequencing | *gag* | 1748–1768 |
| **Gag C_R** | 5’- TCT AAT ACT GTA TCA TCT GC - 3’ | PCR (SGA) and Sequencing | *gag* | 2334–2356 |
| **Gag D_F** | 5’ -TCT CTA GCA GTG GCG CCC G- 3’ | PCR (SGA) | *gag* | 626–644 |
| **Gag D_R** | 5’ -AAT TCC TAT CAT TTT TGG- 3’ | PCR (SGA) | *gag* | 2382-2402. |

**Supplementary Figure 1. Phylogenetic trees and compartmentalisation analysis of HIV-1 *env* and *gag* sequences for participant 0011.** Sequences for participant 0011 were phylogenetically analysed and used to perform statistical analysis (Hyphy) to determine compartmentalisation. Phylogenetic trees were constructed from *env* (panel A), and *gag* (panel B) single genome sequences derived from different compartments. I) Phylogenetic tree showing relatedness of sequences from all compartments, II) PBMC RNA and LN DNA, III) PBMC DNA and LN DNA, IV) LN DNA and plasma, V) PBMC DNA and PBMC RNA, VI) PBMC RNA and plasma, VII) PBMC DNA and plasma. Red circles represent PBMC DNA, open red circles represent PBMC RNA, blue circles represent LN DNA, open blue circles represent LN RNA and yellow circles represent plasma. The number of SGAs generated per compartment are shown in brackets in the key. Statistically significant results (significant F-statistic [FST] and/or Slatkin-Madison [SM] tests) are shown with a red asterisk (*) with accompanying p-values in red. Defective sequences with internal stop codons that are not hypermutants are shown with a black asterisk (*) and those that are hypermutants are shown with a hash (#). Hypermutants were identified by using the Hypermut 2.0 tool available at [www.hiv.lanl.gov](http://www.hiv.lanl.gov) (Fisher’s exact p < 0.05)

*

*

*

*

*

#

#

#

#


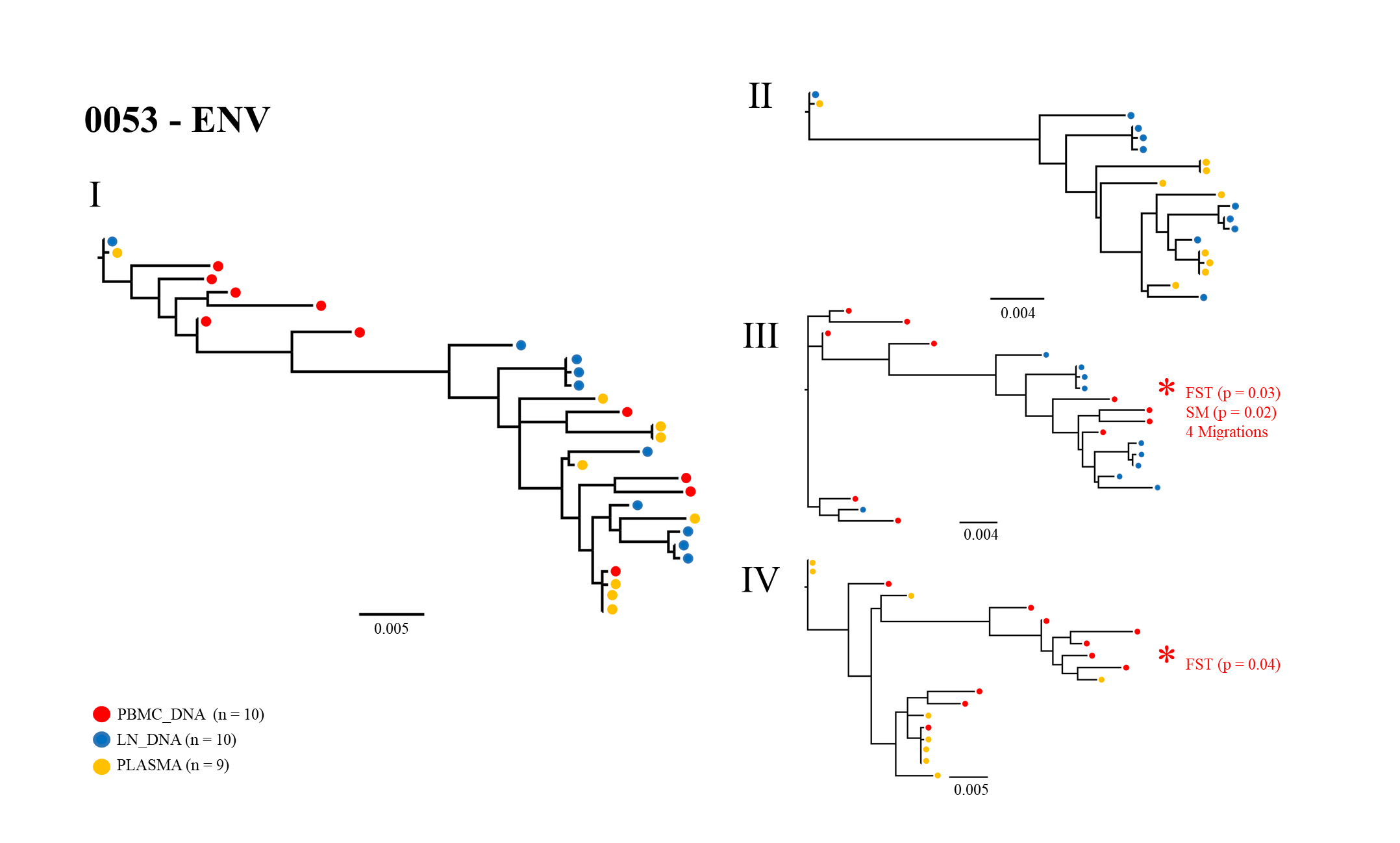


**Supplementary Figure 2. Phylogenetic trees and compartmentalisation analysis of HIV-1 *en*v sequences for participant 0053.** Sequences for participant 0053 were phylogenetically analysed and used to perform statistical analysis (Hyphy) to determine the presence of compartmentalisation. Phylogenetic trees were constructed from *env* single genome sequences derive from different compartments. I) Phylogenetic tree showing relatedness of sequences derived from all compartments, II) LN DNA and plasma, III) PBMC DNA and LN DNA, IV) PBMC DNA and plasma. Red circles represent PBMC DNA, blue circles represent LN DNA, and yellow circles represent plasma. The number of SGAs generated per compartment are shown in brackets in the key. Statistically significant results (significant F-statistic [FST] and/or Slatkin-Madison [SM] tests) are shown with an asterisk (*) with accompanying p-values in red.

#

#

#

**Supplementary Figure 3. Phylogenetic tree and compartmentalisation analysis of HIV-1 *env* and *gag* sequences for participant 079.** Sequences for participant 079 were phylogenetically analysed and used to perform statistical analysis (Hyphy) to determine the presence of compartmentalisation. Phylogenetic trees were constructed from *env* (panel A), and *gag* (panel B) single genome sequences derived from different compartments. I) Phylogenetic tree showing relatedness of sequences derived from all compartments, II) LN DNA and plasma, III) PBMC DNA and LN DNA, IV) PBMC DNA and plasma. Red circles represent PBMC DNA, blue circles represent LN DNA, and yellow circles represent plasma. The number of SGAs generated per compartment are shown in brackets in the key. Statistically significant results (significant F-statistic [FST] and/or Slatkin-Madison [SM] tests) are shown with an asterisk (*) with accompanying p-values in red. Defective sequences that are hypermutants are shown with a hash (#). Hypermutants were identified by using the Hypermut 2.0 tool available at [www.hiv.lanl.gov](http://www.hiv.lanl.gov) (Fisher’s exact p < 0.05).

**Supplementary Figure 4. Phylogenetic trees and compartmentalisation analysis of HIV-1 *gag* sequences for participant 0108.** Sequences for participant 0108 were phylogenetically analysed and used to perform statistical analysis (Hyphy) to determine the presence of compartmentalisation. Phylogenetic trees were constructed from *gag* single genome sequences derived from different compartments. I) Phylogenetic tree showing relatedness of sequences derived from all compartments, II) LN DNA and plasma, III) PBMC DNA and LN DNA, IV) PBMC DNA and plasma. Red circles represent PBMC DNA, blue circles represent LN DNA, and yellow circles represent plasma. The number of SGAs generated per compartment are shown in brackets in the key. Statistically significant results (significant F-statistic [FST] and/or Slatkin-Madison [SM] tests) are shown with an asterisk (*) with accompanying p-values in red.

**Supplementary Figure 5. Phylogenetic trees and compartmentalisation analysis of HIV-1 *env* and *gag* sequences for participant 118.** Sequences for participant 118 were phylogenetically analysed and used to perform statistical analysis (Hyphy) to determine the presence of compartmentalisation. Phylogenetic trees were constructed from *env* (panel A), and *gag* (panel B) single genome sequences derived from PBMC_DNA and plasma. Red circles represent PBMC_DNA and yellow circles represent plasma. The number of SGAs generated per compartment are shown in brackets in the key. Statistically significant results (significant F-statistic [FST] and/or Slatkin-Madison [SM] tests) are shown with a red asterisk (*) with accompanying p-values in red. Defective sequences with internal stop codons that are not hypermutants are shown with a black asterisk (*). The Hypermut 2.0 tool available at [www.hiv.lanl.gov](http://www.hiv.lanl.gov) was used to test for hypermutants (Fisher’s exact p < 0.05)

*

#

#

#

#

#

#

**Supplementary Figure 6. Phylogenetic trees and compartmentalisation analysis of HIV-1 *env* and *gag* sequences for participant 3011.** Sequences for participant 3011 were phylogenetically analysed and used to perform statistical analysis (Hyphy) to determine the presence of compartmentalisation. Phylogenetic trees were constructed from *env* (panel A), and *gag* (panel B) single genome sequences derived from different compartments. I) Phylogenetic tree showing relatedness of sequences derived from all compartments, II) LN DNA and plasma, III) PBMC DNA and LN DNA, IV) PBMC DNA and plasma. Red circles represent PBMC DNA, blue circles represent LN DNA, and yellow circles represent plasma. The number of SGAs generated per compartment are shown in brackets in the key. Statistically significant results (significant F-statistic [FST] and/or Slatkin-Madison [SM] tests) are shown with an asterisk (*) with accompanying p-values in red. Defective sequences that are hypermutants are shown with a hash (#). Hypermutants were identified by using the Hypermut 2.0 tool available at [www.hiv.lanl.gov](http://www.hiv.lanl.gov) (Fisher’s exact p < 0.05).

#

#

#

#

#

#

#

#

#

#

#

#

#

**Supplementary Figure 7. Phylogenetic trees and compartmentalisation analysis of HIV-1 *env* and *gag* sequences for participant 093.** Sequences for participant 093 were phylogenetically analysed and used to perform statistical analysis (Hyphy) to determine the presence of compartmentalisation. Phylogenetic trees were constructed from *env* (panel A), and *gag* (panel B) single genome sequences derived from different compartments. I) Phylogenetic tree showing relatedness of sequences derived from all compartments, II) LN DNA and plasma, III) PBMC DNA and LN DNA, IV) PBMC DNA and plasma. Red circles represent PBMC DNA, blue circles represent LN DNA, and yellow circles represent plasma. The number of SGAs generated per compartment are shown in brackets in the key. Statistically significant results (significant F-statistic [FST] and/or Slatkin-Madison [SM] tests) are shown with an asterisk (*) with accompanying p-values in red. Defective sequences that are hypermutants are shown with a hash (#). Hypermutants were identified by using the Hypermut 2.0 tool available at [www.hiv.lanl.gov](http://www.hiv.lanl.gov) (Fisher’s exact p < 0.05).

#

**Supplementary Figure 8. Phylogenetic trees and compartmentalisation analysis of HIV-1 *gag* sequences for participant 1012.** Sequences for participant 1012 were phylogenetically analysed and used to perform statistical analysis (Hyphy) to determine the presence of compartmentalisation. Phylogenetic trees were constructed from *gag* single genome sequences derived from different compartments. I) Phylogenetic tree showing relatedness of sequences derived from all compartments, II) LN DNA and plasma, III) PBMC DNA and LN DNA, IV) PBMC DNA and plasma. Red circles represent PBMC DNA, blue circles represent LN DNA, and yellow circles represent plasma. The number of SGAs generated per compartment are shown in brackets in the key. Statistically significant results (significant F-statistic [FST] and/or Slatkin-Madison [SM] tests) are shown with a red asterisk (*) with accompanying p-values in red. Defective sequences with internal stop codons that are not hypermutants are shown with a black asterisk (*) and those that are hypermutants are shown with a hash (#). Hypermutants were identified by using the Hypermut 2.0 tool available at [www.hiv.lanl.gov](http://www.hiv.lanl.gov) (Fisher’s exact p < 0.05)

#

#

#

#

*

#

#

*

#

#

*

0.00007


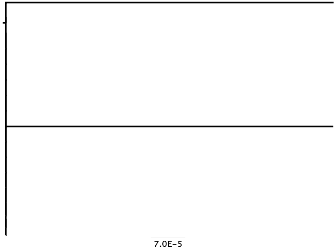


0.0002

0.0004

0.0004

2


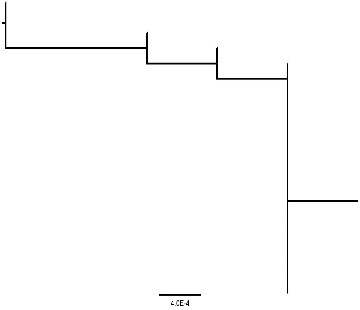

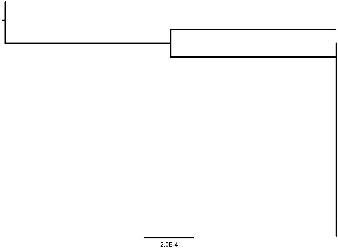

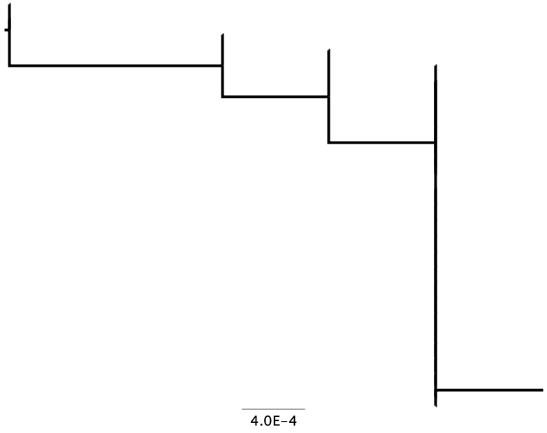

**Supplementary Figure 9. Phylogenetic trees and compartmentalisation analysis of HIV-1 *gag* sequences for participant 1210.** Sequences for participant 1210 were phylogenetically analysed and used to perform statistical analysis (Hyphy) to determine the presence of compartmentalisation. Phylogenetic trees were constructed from *gag* single genome sequences derived from different compartments. I) Phylogenetic tree showing relatedness of sequences derived from all compartments, II) LN DNA and plasma, III) PBMC DNA and LN DNA, IV) PBMC DNA and plasma. Red circles represent PBMC DNA, blue circles represent LN DNA, and yellow circles represent plasma. The number of SGAs generated per compartment are shown in brackets in the key. Statistically significant results (significant F-statistic [FST] and/or Slatkin-Madison [SM] tests) are shown with an asterisk (*) with accompanying p-values in red.

| **PID** | **Compartment** | | **Sequence**  **frequency** | | **197** | **234** | **262** | **275** | **276** | **277** | **278** | **279** | **280** | **281** | **282** | **365** | **371** | **372** | **425** | **429** | **455** | **456** | **457** | **458** | **459** | **461** | **462** | **463** | **471** | **474** |
| --- | --- | --- | --- | --- | --- | --- | --- | --- | --- | --- | --- | --- | --- | --- | --- | --- | --- | --- | --- | --- | --- | --- | --- | --- | --- | --- | --- | --- | --- | --- |
|  |  |  | **HXB2** | | **N** | **N** | **N** | **V** | **N** | **F** | **T** | **D** | **N** | **A** | **K** | **S** | **I** | **V** | **N** | **K** | **T** | **R** | **D** | **G** | **G** | **S** | **N** | **N** | **G** | **D** |
|  |  |  | **CON C** | | **N** | **N** | **N** | **E** | **N** | **L** | **T** | **N** | **N** | **A** | **K** | **S** | **I** | **T** | **N** | **E** | **T** | **R** | **D** | **G** | **G** | **N** | **N** | **T** | **G** | **D** |
| **0011** | | **Plasma** | | **4/8** | . | . | . | E | **T** | L | . | N | . | . | . | . | . | T | . | E | **V** | . | . | . | . | **T** | **D** | **N** | . | . |
|  |  |  |  | **2/8** | . | . | . | E | **T** | L | . | N | . | . | . | . | . | T | . | E | **V** | . | . | . | . | **T** | **D** | **N** | . | **N** |
|  |  |  |  | **1/8** | . | . | . | E | **T** | L | . | N | . | . | . | . | . | T | . | E | **V** | . | . | . | . | **I** | **D** | **N** | . | . |
|  |  |  |  | **1/8** | . | . | . | E | **T** | L | . | N | . | . | . | . | . | T | . | E | **V** | . | . | . | . | **T** | **D** | **N** | **S** | **N** |
|  |  | **LN DNA** | | **4/10** | . | . | . | E | **T** | L | . | N | . | . | . | . | . | T | . | E | **V** | . | . | . | . | **T** | **D** | **N** | . | . |
|  |  |  |  | **3/10** | . | **D** | . | E | **T** | L | . | N | . | **I** | **N** | . | **V** | T | . | E | . | . | . | . | . | **T** | **D** | **N** | . | **N** |
|  |  |  |  | **2/10** | . | . | . | E | **T** | L | . | N | . | . | . | . | . | T | . | E | **V** | . | . | . | . | **T** | **D** | **N** | . | **N** |
|  |  |  |  | **1/10** | . | . | . | E | **T** | L | . | N | . | . | . | . | . | T | . | E | **V** | . | . | . | . | **T** | **D** | **N** | **S** | **N** |
|  |  | **LN RNA** | | **4/5** | . | . | . | E | **T** | L | . | N | . | . | . | . | . | T | . | E | **V** | . | . | . | . | **T** | **D** | **N** | . | . |
|  |  |  |  | **1/5** | . | . | . | E | **T** | L | . | N | . | . | . | . | . | T | . | E | **V** | . | . | . | . | **T** | **D** | **N** | **S** | **N** |
|  |  | **PBMC DNA** | | **6/10** | . | . | . | E | **T** | L | . | N | . | . | . | . | . | T | . | E | **V** | . | . | . | . | **T** | **D** | **N** | . | . |
|  |  |  |  | **2/10** | . | . | . | E | **T** | L | . | N | . | . | . | . | . | T | . | E | **V** | . | . | . | . | **T** | **D** | **N** | . | **N** |
|  |  |  |  | **1/10** | . | . | . | E | **T** | L | . | N | . | . | . | . | . | T | . | E | **V** | . | . | . | . | **T** | . | **N** | . | . |
|  |  |  |  | **1/10** | . | . | . | E | **T** | L | . | N | . | . | . | . | . | T | . | E | **V** | . | . | . | . | **K** | **D** | **N** | . | . |
|  |  | **PBMC RNA** | | **6/10** | . | **D** | . | E | **T** | L | . | N | . | **I** | **N** | . | **V** | T | . | E | . | . | . | . | . | **T** | **D** | **N** | . | **N** |
|  |  |  |  | **3/10** | . | . | . | E | **T** | L | . | N | . | . | . | . | . | T | . | E | **V** | . | . | . | . | **T** | **D** | **N** | **S** | **N** |
|  |  |  |  | **1/10** | . | . | . | E | **T** | L | . | N | . | . | **N** | . | . | T | . | E | **V** | . | . | . | . | **T** | **D** | **N** | . | . |
| **0053** | | **Plasma** | | **5/9** | . | . | . | E | . | L | . | **D** | . | **V** | . | . | . | T | . | **G** | . | . | . | . | . | N | **S** | **S** | **I** | **N** |
|  |  |  |  | **2/9** | . | . | . | E | . | L | . | **D** | . | **V** | . | . | . | T | . | **G** | . | . | . | . | . | N | **S** | **S** | **I** | **N** |
|  |  |  |  | **1/9** | . | **S** | . | E | . | L | . | **D** | . | **V** | . | . | . | T | . | **G** | . | . | . | . | . | N | **S** | **S** | **I** | **N** |
|  |  |  |  | **1/9** | . | . | . | E | . | L | . | N | . | **V** | . | . | . | T | . | **G** | . | . | . | . | . | N | **R** | T | **I** | **N** |
|  |  | **LN** | | **5/10** | . | . | . | E | . | L | . | **D** | . | **V** | . | . | . | T | . | **G** | . | . | . | . | . | N | **S** | **S** | **I** | **N** |
|  |  |  |  | **4/10** | . | **S** | . | E | . | L | . | **D** | . | **V** | . | . | . | T | . | **G** | . | . | . | . | . | N | **S** | **S** | **I** | **N** |
|  |  |  |  | **1/10** | . | . | . | E | . | L | . | N | . | **V** | . | . | . | T | . | **G** | . | . | . | . | . | N | **R** | T | **I** | **N** |
|  |  | **PBMC** | | **3/10** | . | . | . | E | . | L | . | **D** | . | **V** | . | . | . | T | . | **G** | . | . | . | . | . | N | **S** | **S** | **I** | **N** |
|  |  |  |  | **2/10** | . | . | . | E | . | L | . | N | . | **V** | . | . | . | T | . | **G** | . | . | . | . | . | N | **G** | T | **I** | **N** |
|  |  |  |  | **1/10** | . | . | . | E | . | L | . | **D** | . | **V** | . | . | . | T | . | **G** | . | . | . | . | . | **T** | **S** | **S** | **I** | **N** |
|  |  |  |  | **1/10** | . | . | . | E | . | L | . | N | . | **V** | . | . | . | T | . | **G** | . | . | . | . | . | N | **R** | T | **I** | **N** |
|  |  |  |  | **1/10** | . | . | . | E | . | L | . | N | . | **V** | . | . | . | T | . | **G** | . | . | . | . | . | N | **S** | T | **T** | **N** |
|  |  |  |  | **1/10** | . | . | . | E | . | L | **A** | N | . | **V** | . | . | . | T | . | **G** | . | . | . | . | . | N | **G** | T | **I** | **N** |
|  |  |  |  | **1/10** | . | . | . | E | . | L | . | N | . | **V** | . | . | . | T | . | **G** | . | . | . | . | . | N | **S** | - | **T** | **N** |
| **079** | | **Plasma** | | **4/7** | . | . | . | E | . | **M** | . | N | . | . | . | . | . | T | . | **Q** | . | . | . | . | . | N | **T** | **N** | . | . |
|  |  |  |  | **1/7** | . | . | . | E | . | **I** | . | N | . | . | . | . | . | T | . | **Q** | . | . | . | . | . | N | **T** | **N** | . | . |
|  |  |  |  | **1/7** | . | . | . | E | . | **I** | . | N | . | . | . | . | . | T | . | **Q** | . | . | . | . | . | **G** | **T** | **N** | . | . |
|  |  |  |  | **1/7** | . | . | . | E | . | **M** | . | N | . | . | . | . | . | **K** | . | **Q** | . | . | . | . | . | N | **T** | **N** | . | . |
|  |  | **LN** | | **3/8** | . | . | . | E | . | **M** | . | N | . | . | . | . | . | T | . | **Q** | . | . | . | . | . | N | **T** | **N** | . | . |
|  |  |  |  | **2/8** | . | . | . | E | . | **M** | . | N | . | . | . | . | . | **K** | . | **Q** | . | . | . | . | . | N | **T** | **N** | . | . |
|  |  |  |  | **1/8** | . | . | . | E | . | **I** | . | N | . | . | . | . | . | T | . | **Q** | . | . | . | . | . | N | **T** | **N** | . | . |
|  |  |  |  | **1/8** | . | . | . | E | . | **M** | . | N | . | . | . | . | **V** | T | . | **Q** | . | . | . | . | . | N | **T** | **N** | . | . |
|  |  |  | | **1/8** | . | . | . | E | . | **I** | . | N | . | . | . | . | . | T | . | **Q** | . | **K** | . | **R** | **R** | N | **T** | **N** | **R** | . |
|  |  | **PBMC** | | **3/12** | . | . | . | E | . | **M** | . | N | . | . | . | . | . | T | . | **Q** | . | . | . | . | . | N | **T** | **N** | . | . |
|  |  |  |  | **2/12** | . | . | . | E | . | **I** | . | N | . | . | . | . | . | T | . | **Q** | . | . | . | . | . | N | **T** | **N** | . | . |
|  |  |  |  | **2/12** | . | . | . | E | . | **M** | . | N | . | . | . | . | . | **K** | . | **Q** | . | . | . | . | . | N | **T** | **N** | . | . |
|  |  |  |  | **1/12** | . | . | . | E | . | **M** | . | N | . | . | . | . | . | **R** | . | **Q** | . | . | . | . | . | N | **T** | **N** | . | . |
|  |  |  |  | **1/12** | . | . | . | E | . | **M** | . | N | . | . | . | . | . | T | . | **Q** | . | . | . | . | . | N | **T** | **N** | . | . |
|  |  |  |  | **1/12** | . | . | . | E | . | **I** | . | N | . | . | . | . | . | T | . | **Q** | . | . | . | . | . | N | **T** | **N** | . | . |
|  |  |  |  | **1/12** | . | . | . | E | . | **I** | . | N | . | . | . | . | . | T | . | **Q** | . | . | . | . | . | **G** | **T** | **N** | . | . |
|  |  |  |  | **1/12** | . | . | . | E | . | **I** | . | N | . | . | . | . | . | **K** | . | **Q** | . | . | . | . | . | **T** | **T** | **N** | . | . |
| **118** | | **Plasma** | | **5/8** | . | . | . | E | . | L | . | N | . | . | . | . | . | T | . | **G** | . | . | . | . | . | **S** | **E** | **N** | **A** | D |
|  |  |  |  | **2/8** | . | . | . | E | . | L | . | N | . | . | . | . | . | T | . | **G** | . | . | . | . | . | N | **E** | **N** | **A** | D |
|  |  |  |  | **1/8** | . | . | . | E | . | L | . | N | . | . | . | . | . | T | . | **G** | . | . | . | . | . | **S** | **D** | **N** | **A** | D |
|  |  | **PBMC** | | **4/9** | . | . | . | E | D | L | . | N | . | . | . | . | . | T | . | **G** | . | . | . | . | . | **-** | **E** | **N** | **A** | D |
|  |  |  |  | **4/9** | . | . | . | E | . | L | . | N | . | . | . | . | . | T | . | **G** | . | . | . | . | . | **S** | **E** | **N** | **A** | D |
|  |  |  |  | **1/9** | . | . | . | E | D | L | . | N | . | . | . | . | . | T | . | **G** | . | . | . | . | . | **-** | **Q** | **E** | **A** | **N** |
| **3011** | | **Plasma** | | **5/9** | . | . | . | E | . | L | . | **D** | . | . | . | . | . | **K** | . | **G** | . | . | . | . | . | **S** | . | **Q** | . | **N** |
|  |  |  |  | **2/9** | . | . | . | E | . | L | . | **D** | . | **T** | . | . | . | **R** | . | **G** | . | . | . | . | . | **S** | . | **Q** | . | **N** |
|  |  |  |  | **1/9** | . | . | . | E | . | L | . | **D** | . | . | . | . | . | **R** | . | **G** | . | . | . | . | . | **S** | . | **Q** | . | **N** |
|  |  |  |  | **1/9** | . | . | . | E | . | L | . | **D** | . | . | . | **A** | . | **K** | . | **G** | . | . | . | . | . | **S** | . | **Q** | . | **N** |
|  |  | **LN** | | **3/8** | . | . | . | E | . | L | . | **D** | . | . | . | . | . | **R** | . | **G** | . | . | . | . | . | **S** | . | **Q** | . | **N** |
|  |  |  |  | **2/8** | . | . | . | E | . | L | . | **D** | . | . | . | **A** | . | **K** | . | **G** | . | . | . | . | . | **S** | . | **Q** | . | **N** |
|  |  |  |  | **1/8** | . | . | . | E | . | L | . | **D** | . | . | . | . | . | **R** | . | **R** | . | . | . | . | . | **S** | . | **Q** | . | **N** |
|  |  |  |  | **1/8** | . | . | . | E | . | L | . | **D** | . | . | . | **A** | . | **K** | . | **R** | . | . | . | . | **E** | **S** | . | **Q** | **R** | **N** |
|  |  |  |  | **1/8** | . | . | . | E | . | L | . | **D** | . | **T** | . | . | . | **R** | . | **G** | . | . | . | . | . | **S** | . | **Q** | . | **N** |
|  |  | **PBMC** | | **4/10** | . | . | . | E | . | L | . | **D** | . | . | . | **A** | . | **K** | . | **G** | . | . | . | . | . | **S** | . | **Q** | . | **N** |
|  |  |  |  | **2/10** | . | . | . | E | . | L | . | **D** | . | **T** | . | . | . | **R** | . | **G** | . | . | . | . | . | **S** | . | **Q** | . | **N** |
|  |  |  |  | **1/10** | . | . | . | E | . | L | . | **D** | . | . | . | . | . | **R** | . | **G** | . | . | . | . | . | **S** | . | **Q** | . | **N** |
|  |  |  |  | **1/10** | . | . | . | E | . | L | . | **D** | . | **T** | . | **A** | . | **K** | . | **G** | . | . | . | . | . | **S** | . | **Q** | **E** | **N** |
|  |  |  |  | **1/10** | . | . | . | E | . | L | . | **D** | . | . | . | **A** | . | **K** | . | **G** | . | . | . | . | . | **S** | . | **Q** | **E** | **N** |
|  |  |  |  | **1/10** | . | . | . | E | . | L | . | **D** | . | . | . | . | . | **K** | . | **G** | . | . | . | . | . | **G** | . | **Q** | **E** | **N** |
| **093** | | **Plasma** | | **10/10** | . | . | . | E | . | **I** | . | **D** | . | . | . | . | . | T | . | **K** | **L** | . | . | . | . | **T** | **T** | T | . | **N** |
|  |  | **LN** | | **7/7** | . | . | . | E | . | **I** | . | **D** | . | . | . | . | . | T | . | **K** | **L** | . | . | . | . | **T** | **T** | T | . | **N** |
|  |  | **PBMC** | | **7/9** | . | . | . | E | . | **I** | . | **D** | . | . | . | . | . | T | . | **K** | **L** | . | . | . | . | **T** | **T** | T | . | **N** |
|  |  |  |  | **2/9** | . | . | . | E | . | **I** | . | **D** | . | . | . | . | . | T | . | **K** | **L** | . | . | . | . | **T** | **T** | T | **R** | **N** |

**Supplementary Figure 10. bnAb escape mutations from epitopes in the CD4 binding region.** Mutations that were different from the consensus C sequence (http://www.hiv.lanl.gov/) are shown in red font. Sites where the percentage of mutations present in one compartment is at least 50% different to another are shown in red blocks and expanded on in Table 3.5. Sequence frequency shows the number of times that specific sequence shows up in each compartment. HXB2 is the reference sequence that is used to number the amino acids in the participant alignments. Con C is the consensus C sequence from the HIV Los Alamos sequence database.

| **PID** | **Compartment** | **Sequence**  **Frequency** | **130** | **156** | **160** | **161** | **162** | **163** | **`165** | **166** | **167** | **168** | **169** | **170** | **171** | **173** | **197** | **332** |
| --- | --- | --- | --- | --- | --- | --- | --- | --- | --- | --- | --- | --- | --- | --- | --- | --- | --- | --- |
|  |  | **HXB2** | **K** | **N** | **N** | **I** | **S** | **T** | **I** | **R** | **G** | **K** | **V** | **Q** | **K** | **Y** | **N** | **N** |
|  |  | **CON C** | **N** | **N** | **N** | **I** | **T** | **T** | **L** | **R** | **D** | **K** | **K** | **Q** | **K** | **Y** | **N** | **N** |
| **0011** | **Plasma** | **3/8** | N | . | . | **T** | T | . | I | . | D | . | K | Q | **Q** | . | . | . |
|  |  | **2/8** | **S** | . | . | **T** | T | . | I | . | D | . | K | Q | **Q** | . | . | . |
|  |  | **1/8** | **K** | . | . | **T** | T | . | I | . | D | . | K | Q | **Q** | . | . | . |
|  |  | **1/8** | **K** | . | . | **T** | T | . | I | **G** | D | . | K | Q | **Q** | . | . | . |
|  |  | **1/8** | **H** | . | . | **M** | T | . | **L** | . | D | . | **Q** | **K** | . | . | . | . |
|  | **LN DNA** | **2/10** | N | . | . | **T** | T | . | I | . | D | . | K | Q | **Q** | . | . | . |
|  |  | **1/10** | N | . | . | **T** | T | . | I | . | **G** | . | K | Q | **Q** | . | . | . |
|  |  | **1/10** | **K** | . | . | **T** | T | . | I | . | D | . | K | Q | **Q** | . | . | . |
|  |  | **1/10** | N | . | **T** | **T** | **A** | . | I | **K** | **G** | . | K | **K** | **E** | . | . | . |
|  |  | **1/10** | N | . | **T** | **T** | **A** | **I** | I | **K** | D | . | K | **K** | **E** | . | . | . |
|  |  | **1/10** | N | . | . | **T** | T | . | I | . | D | . | K | Q | **Q** | . | . | . |
|  |  | **1/10** | **K** | . | . | **T** | T | . | I | **G** | D | . | K | Q | **Q** | . | . | . |
|  |  | **1/10** | **H** | . | . | **M** | T | . | **L** | . | D | . | **Q** | **K** | . | . | . | . |
|  |  | **1/10** | N | . | **T** | **T** | **A** | . | I | **K** | D | . | K | Q | **E** | . | . | . |
|  | **LN RNA** | **3/5** | N | . | . | **T** | T | . | I | . | D | . | K | Q | **Q** | . | . | . |
|  |  | **1/5** | N | . | . | **T** | T | . | I | . | **G** | . | K | Q | **Q** | . | . | . |
|  |  | **1/5** | **K** | . | . | **T** | T | . | I | **G** | D | . | K | Q | **Q** | . | . | . |
|  | **PBMC DNA** | **5/10** | N | . | . | **T** | T | . | I | . | D | . | K | Q | **Q** | . | . | . |
|  |  | **3/10** | N | . | . | **M** | T | . | **L** | . | D | . | **Q** | **K** | . | . | . | . |
|  |  | **1/10** | N | . | . | **M** | T | . | **L** | . | D | . | **Q** | **K** | . | . | . | . |
|  |  | **1/10** | **K** | . | . | **T** | T | . | I | . | D | . | K | Q | **Q** | . | . | . |
|  | **PBMC RNA** | **2/10** | N | . | **T** | **T** | **A** | . | I | **K** | D | . | K | Q | **E** | . | . | . |
|  |  | **2/10** | N | . | **T** | **T** | **A** | . | I | **K** | **G** | . | K | Q | **E** | . | . | . |
|  |  | **1/10** | N | . | . | **T** | T | . | I | . | D | . | K | Q | **Q** | . | . | . |
|  |  | **1/10** | N | . | . | **T** | T | . | I | . | **G** | . | K | Q | **Q** | . | . | ***** |
|  |  | **1/10** | N | . | **T** | **T** | **A** | . | I | **K** | **G** | . | K | Q | **E** | . | . | . |
|  |  | **1/10** | N | . | **T** | **T** | **A** | . | I | **K** | D | . | K | Q | **E** | . | . | . |
|  |  | **1/10** | N | . | . | **T** | T | . | I | . | **G** | . | K | Q | **Q** | . | . | . |
| **0053** | **Plasma** | **3/9** | **E** | . | . | **A** | T | . | I | **K** | D | . | K | **R** | **R** | . | . | . |
|  |  | **2/9** | **E** | . | . | **A** | T | . | I | . | D | . | K | **R** | . | . | . | . |
|  |  | **1/9** | **E** | . | . | **A** | T | . | I | . | D | . | K | **R** | . | . | . | . |
|  |  | **1/9** | **E** | . | . | **A** | T | . | I | . | D | . | **I** | **R** | . | . | . | . |
|  |  | **1/9** | **E** | . | . | **A** | T | . | I | . | D | **R** | K | **R** | . | . | . | . |
|  |  | **1/9** | **E** | . | . | **A** | T | . | I | . | D | . | K | **R** | **R** | . | . | . |
|  | **LN** | **4/10** | **E** | . | . | **A** | T | . | I | . | D | . | K | **R** | **R** | . | . | . |
|  |  | **3/10** | **E** | . | . | **A** | T | . | I | . | D | . | **A** | **R** | . | . | . | . |
|  |  | **1/10** | **E** | . | . | **A** | T | . | I | . | D | . | **I** | **R** | . | . | . | . |
|  |  | **01/10** | **E** | . | . | **A** | T | . | I | . | D | **R** | K | **R** | . | . | . | . |
|  |  | **1/10** | **E** | . | . | **A** | T | . | I | **K** | D | . | K | **R** | **R** | . | . | . |
|  | **PBMC** | **4/10** | **E** | . | . | **A** | T | . | I | . | D | **R** | K | **R** | . | . | . | . |
|  |  | **4/10** | **E** | . | . | **A** | T | . | I | . | D | . | K | **R** | . | . | . | . |
|  |  | **1/10** | **E** | . | . | **A** | T | . | I | . | D | . | K | **R** | . | . | . | . |
| **079** | **Plasma** | **2/7** | N | . | . | **V** | T | . | **V** | . | D | . | **E** | **K** | . | . | . | . |
|  |  | **2/7** | **S** | . | . | **V** | T | . | I | . | D | . | **E** | **K** | . | . | . | . |
|  |  | **1/7** | N | . | . | **V** | T | . | **V** | . | D | . | **E** | **K** | . | . | . | . |
|  |  | **1/7** | N | . | . | **V** | T | . | **V** | . | D | . | **E** | **K** | . | . | . | . |
|  |  | **1/7** | N | . | . | **V** | T | . | I | . | D | . | **E** | **K** | . | . | . | . |
|  | **LN** | **2/8** | N | . | . | **V** | T | . | I | . | D | . | **E** | **K** | . | . | . | . |
|  |  | **2/8** | N | . | . | **V** | T | . | **V** | . | D | . | **E** | **K** | . | . | . | . |
|  |  | **1/8** | N | . | . | **V** | T | . | **L** | . | D | . | **E** | **K** | . | . | . | . |
|  |  | **1/8** | N | . | . | **V** | T | . | **V** | . | D | . | **E** | **K** | . | . | . | . |
|  |  | **1/8** | **S** | . | . | **V** | T | . | I | . | D | . | **E** | **K** | . | . | . | . |
|  |  | **1/8** | N | . | . | **V** | T | . | **V** | . | D | . | **E** | **K** | . | . | . | . |
|  | **PBMC** | **3/12** | N | . | . | **V** | T | . | **V** | . | D | . | **E** | **K** | . | . | . | . |
|  |  | **2/12** | N | . | . | **V** | T | . | **V** | . | D | . | **E** | **K** | . | . | . | . |
|  |  | **2/12** | **S** | . | . | **V** | T | . | I | . | D | . | **E** | **K** | . | . | . | . |
|  |  | **2/12** | N | . | . | **V** | T | . | I | . | D | . | **E** | **K** | . | . | . | . |
|  |  | **1/12** | N | . | . | **V** | T | . | **V** | . | D | . | **E** | **K** | . | . | . | . |
|  |  | **1/12** | N | . | . | **V** | T | . | **V** | . | D | . | **E** | **K** | . | . | . | . |
|  |  | **1/12** | N | . | . | **V** | T | . | **V** | . | D | . | **E** | **K** | . | . | . | . |
| **118** | **Plasma** | **4/8** | **T** | . | . | . | T | . | **L** | . | D | . | **N** | **K** | **Q** | . | . | . |
|  |  | **2/8** | **T** | . | . | . | T | . | **L** | . | D | . | **N** | **K** | **Q** | . | . | . |
|  |  | **1/8** | **T** | . | . | . | T | . | **L** | . | D | . | **S** | **K** | **Q** | . | . | . |
|  | **PBMC** | **3/9** | **T** | . | . | . | T | . | **L** | . | D | . | **R** | **K** | **Q** | . | . | . |
|  |  | **3/9** | **T** | . | . | . | T | . | **L** | . | D | . | **N** | **K** | **Q** | . | . | . |
|  |  | **1/9** | **T** | . | . | . | T | . | **L** | . | D | . | K | **K** | **Q** | . | . | . |
|  |  | **1/9** | **T** | . | . | . | T | . | **L** | . | D | . | **R** | **K** | **Q** | . | . | . |
|  |  | **1/9** | **T** | . | . | **V** | T | . | **L** | . | D | . | K | **K** | **Q** | . | . | . |
| **3011** | **Plasma** | **9/9** | N | . | . | **T** | T | . | I | **K** | D | **R** | K | . | . | **H** | . | . |
|  | **LN** | **7/8** | N | . | . | **T** | T | . | I | **K** | D | **R** | K | . | . | **H** | . | . |
|  |  | **1/8** | N | . | . | **T** | T | . | I | **K** | D | . | K | . | . | **H** | . | . |
|  | **PBMC** | **10/10** | N | . | . | **T** | T | . | I | **K** | D | **R** | K | . | . | **H** | . | . |
| **093** | **Plasma** | **7/10** | N | . | . | **T** | T | . | I | . | D | . | K | . | . | . | . | . |
|  |  | **1/10** | N | . | . | **T** | T | . | I | . | D | . | **E** | . | . | . | . | . |
|  |  | **1/10** | N | . | . | **T** | T | . | I | . | D | . | **Q** | . | . | . | . | . |
|  |  | **1/10** | N | **K** | . | **T** | T | . | I | . | D | . | **Q** | . | . | . | . | . |
|  | **LN** | **5/7** | N | . | . | **T** | T | . | I | . | D | . | K | . | . | . | . | . |
|  |  | **1/7** | N | . | . | **T** | T | . | I | . | D | . | **E** | . | . | . | . | . |
|  |  | **1/7** | N | . | . | **T** | T | . | I | . | D | . | **N** | . | . | . | . | . |
|  | **PBMC** | **9/9** | N | . | . | **T** | T | . | I | . | D | . | K | . | . | . | . | . |

**Supplementary Figure 11. bnAb escape mutations from epitopes in the V1V2 region.** Mutations that were different from the consensus C sequence (http://www.hiv.lanl.gov/) are shown in red font. Sites where the percentage of mutations present in one compartment is at least 50% different to another are shown in red blocks and expanded on in Table 3.5. Sequence frequency shows the number of times that specific sequence shows up in each compartment. HXB2 is the reference sequence that is used to number the amino acids in the participant alignments. Con C is the consensus C sequence from the HIV Los Alamos sequence database.

| **PID** | **Compartment** | **Sequence**  **frequency** | **165** | **295** | **301** | **304** | **323** | **325** | **328** | **330** | **332** | **334** | **339** | **341** | **343** |
| --- | --- | --- | --- | --- | --- | --- | --- | --- | --- | --- | --- | --- | --- | --- | --- |
|  |  | **HXB2** | **I** | **N** | **N** | **R** | **I** | **N** | **Q** | **H** | **N** | **S** | **N** | **T** | **K** |
|  |  | **CON C** | **L** | **V** | **N** | **R** | **I** | **D** | **Q** | **H** | **N** | **S** | **N** | **T** | **Q** |
| **0011** | **Plasma** | **2/8** | **I** | V | . | . | . | . | **K** | . | . | . | **D** | **A** | Q |
|  |  | **2/8** | **I** | V | . | . | . | . | **K** | . | . | . | **D** | **A** | **H** |
|  |  | **1/8** | **I** | V | . | . | . | . | **K** | . | . | . | **D** | **A** | Q |
|  |  | **1/8** | L | V | . | . | . | **.** | **K** | . | . | . | **D** | **A** | Q |
|  |  | **1/8** | **I** | V | . | . | . | **N** | . | . | . | . | **D** | **A** | Q |
|  |  | **1/8** | **I** | V | . | . | . | **N** | . | . | . | . | **D** | **A** | Q |
|  | **LN DNA** | **2/10** | **I** | V | . | . | . | **.** | **E** | . | . | . | . | **A** | Q |
|  |  | **2/10** | **I** | V | . | . | . | **.** | **K** | . | . | . | **D** | **A** | **H** |
|  |  | **1/10** | **I** | V | . | . | . | **.** | **K** | . | . | . | **D** | **A** | Q |
|  |  | **1/10** | **I** | V | . | . | . | **N** | . | . | . | . | **D** | **A** | Q |
|  |  | **1/10** | **I** | V | **D** | . | . | **N** | . | . | . | . | **D** | **A** | Q |
|  |  | **1/10** | L | V | . | . | . | **.** | **K** | . | . | . | **D** | **A** | Q |
|  |  | **1/10** | **I** | V | . | . | . | **N** | . | . | . | . | **D** | **A** | Q |
|  | **LN RNA** | **3/5** | **I** | V | . | . | . | **N** | . | . | . | . | **D** | **A** | Q |
|  |  | **1/5** | **I** | V | . | . | . | **.** | **K** | . | . | . | **D** | **A** | Q |
|  |  | **1/5** | **I** | V | . | . | . | **.** | **K** | . | . | . | **D** | **A** | Q |
|  | **PBMC DNA** | **2/10** | **I** | V | . | . | . | **N** | . | . | . | . | **D** | **A** | Q |
|  |  | **1/10** | **I** | V | . | . | . | **.** | **K** | . | . | . | **D** | **A** | Q |
|  |  | **1/10** | **I** | V | . | . | . | **N** | . | . | . | . | **D** | **A** | Q |
|  |  | **1/10** | **I** | V | . | . | . | . | **K** | . | . | . | **D** | **A** | **H** |
|  |  | **1/10** | **I** | V | . | . | . | . | **K** | . | . | . | **D** | **A** | **H** |
|  |  | **1/10** | L | V | . | . | . | . | **K** | . | . | . | **D** | **A** | Q |
|  |  | **1/10** | L | V | . | . | . | . | **K** | . | . | . | **D** | **A** | Q |
|  |  | **1/10** | L | V | . | . | . | . | **K** | . | . | . | **D** | **A** | Q |
|  |  | **1/10** | L | V | . | . | . | . | **K** | . | . | . | **D** | . | Q |
|  | **PBMC RNA** | **3/10** | **I** | V | . | . | . | . | **E** | . | . | . | . | **A** | Q |
|  |  | **3/10** | **I** | V | . | . | . | . | **K** | . | . | . | **D** | **A** | **H** |
|  |  | **2/10** | **I** | V | . | . | . | . | **E** | . | . | . | . | **A** | Q |
|  |  | **1/10** | **I** | V | . | **K** | . | . | **E** | . | . | . | . | **A** | Q |
|  |  | **1/10** | **I** | **C** | **Q** | . | **N** | **R** | **K** | **T** | **-** | **-** | **D** | **A** | Q |
| **0053** | **Plasma** | **5/9** | **I** | **K** | . | . | . | . | . | . | . | . | . | . | **E** |
|  |  | **2/9** | **I** | **E** | . | . | . | **N** | . | . | . | . | **E** | . | **E** |
|  |  | **1/9** | **I** | **K** | . | . | . | **.** | . | . | . | . | . | . | **E** |
|  |  | **1/9** | **I** | **E** | . | . | . | **.** | . | . | . | . | . | . | **E** |
|  | **LN** | **3/10** | **I** | **E** | . | . | . | **N** | . | . | . | . | . | . | **E** |
|  |  | **2/10** | **I** | **K** | . | . | . | **.** | . | . | . | . | . | . | **E** |
|  |  | **2/10** | **I** | **K** | . | . | . | **N** | . | . | . | . | . | . | **E** |
|  |  | **1/10** | **I** | **E** | . | . | . | **.** | . | . | . | . | . | . | **E** |
|  |  | **1/10** | **I** | **E** | . | . | . | **.** | . | . | . | . | . | . | **E** |
|  |  | **1/10** | **I** | **K** | . | . | . | **N** | . | . | . | . | . | . | **E** |
|  | **PBMC** | **3/10** | **I** | **E** | . | . | . | **.** | . | . | . | . | . | . | **E** |
|  |  | **2/10** | **I** | **K** | . | . | . | **.** | . | . | . | . | . | . | **E** |
|  |  | **1/10** | **I** | **E** | . | . | . | **.** | . | . | . | . | . | . | **E** |
|  |  | **1/10** | **I** | **E** | . | . | . | **.** | . | . | . | . | . | . | **E** |
|  |  | **1/10** | **I** | **K** | . | . | . | **.** | . | . | . | . | . | . | **D** |
|  |  | **1/10** | **I** | **K** | . | . | . | **N** | . | . | . | . | **E** | . | **E** |
|  |  | **1/10** | **I** | **E** | . | . | . | **N** | . | . | . | . | . | . | **E** |
| **079** | **Plasma** | **4/7** | **I** | V | . | . | . | D | **R** | **Y** | . | . | . | . | Q |
|  |  | **2/7** | **V** | V | . | . | . | D | **R** | **Y** | . | . | . | . | Q |
|  |  | **1/7** | **V** | V | . | . | . | **N** | **K** | **Y** | . | . | . | . | Q |
|  | **LN** | **3/8** | **V** | V | . | . | . | D | **R** | **Y** | . | . | . | . | Q |
|  |  | **3/8** | **I** | V | . | . | . | D | **R** | **Y** | . | . | . | . | Q |
|  |  | **1/8** | L | V | . | . | . | D | **R** | **Y** | . | . | . | . | Q |
|  |  | **1/8** | **V** | V | . | . | . | **N** | **K** | **Y** | . | . | . | . | Q |
|  | **PBMC** | **4/12** | **I** | V | . | . | . | D | **R** | **Y** | . | . | . | . | Q |
|  |  | **4/12** | **V** | V | . | . | . | D | **R** | **Y** | . | . | . | . | Q |
|  |  | **3/12** | **V** | V | . | . | . | **N** | **K** | **Y** | . | . | . | . | Q |
|  |  | **1/12** | **V** | V | . | . | . | D | . | **Y** | . | . | . | . | Q |
| **118** | **Plasma** | **7/8** | L | **T** | . | . | . | **N** | . | . | . | . | . | . | **E** |
|  |  | **1/8** | L | **T** | . | . | . | **N** | . | . | . | . | . | . | **E** |
|  | **PBMC** | **5/9** | L | **T** | . | . | . | D | . | . | . | **A** | . | . | **E** |
|  |  | **3/9** | L | **T** | . | . | . | **N** | . | . | . | . | . | . | **E** |
|  |  | **1/9** | L | **T** | . | . | . | D | . | . | . | **A** | . | . | **E** |
| **3011** | **Plasma** | **8/9** | **I** | V | . | **S** | . | D | . | . | . | . | **E** | . | Q |
|  |  | **1/9** | **.** | V | . | **S** | . | D | . | . | . | . | **E** | . | Q |
|  | **LN** | **8/8** | **I** | V | . | **S** | . | D | . | . | . | . | **E** | . | Q |
|  | **PBMC** | **9/10** | **I** | V | . | **S** | . | D | . | . | . | . | **E** | . | Q |
|  |  | **1/10** | **I** | V | . | **S** | . | D | . | . | . | . | **E** | . | Q |
| **093** | **Plasma** | **8/10** | **I** | **N** | . | . | . | **N** | . | . | . | . | . | . | Q |
|  |  | **1/10** | **I** | **K** | . | . | . | **N** | . | . | . | . | . | . | Q |
|  |  | **1/10** | **I** | **T** | . | . | . | **N** | . | . | . | . | . | . | Q |
|  | **LN** | **2/7** | **I** | **T** | . | . | . | **N** | . | . | . | . | . | . | Q |
|  |  | **2/7** | **I** | **N** | . | . | . | **N** | . | . | . | . | . | . | Q |
|  |  | **2/7** | **I** | **K** | . | . | . | **N** | . | . | . | . | . | . | Q |
|  |  | **1/7** | **I** | **N** | . | . | . | **N** | . | . | . | . | . | . | Q |
|  | **PBMC** | **5/9** | **I** | **N** | . | . | . | **N** | . | . | . | . | . | . | Q |
|  |  | **2/9** | **I** | **T** | . | . | . | **N** | . | . | . | . | . | . | Q |
|  |  | **2/9** | **I** | **N** | . | . | . | **N** | . | . | . | . | . | . | Q |

**Supplementary Figure 12. bnAb escape mutations from epitopes in the V3 region.** Mutations that were different from the consensus C sequence (http://www.hiv.lanl.gov/) are shown in red font. Sites where the percentage of mutations present in one compartment is at least 50% different to another are shown in red blocks and expanded on in Table 3.5. Sequence frequency shows the number of times that specific sequence shows up in each compartment. HXB2 is the reference sequence that is used to number the amino acids in the participant alignments. Con C is the consensus C sequence from the HIV Los Alamos sequence database**.**

**Supplementary Figure 13. bnAb escape mutations from epitopes in the gp120/41 region.** Mutations that were different from the consensus C sequence (http://www.hiv.lanl.gov/) are shown in red font. Sites where the percentage of mutations present in one compartment is at least 50% different to another are shown in red blocks and expanded on in Table 3.5. Sequence frequency shows the number of times that specific sequence shows up in each compartment. HXB2 is the reference sequence that is used to number the amino acids in the participant alignments. Con C is the consensus C sequence from the HIV Los Alamos sequence database.

| **PID** | **Compartment** | **Sequence**  **Frequency** | **671** | **672** | **673** | **674** | **676** | **677** | **680** | **683** |
| --- | --- | --- | --- | --- | --- | --- | --- | --- | --- | --- |
|  |  | **HXB2** | **N** | **W** | **F** | **N** | **T** | **N** | **W** | **K** |
|  |  | **CON C** | **N** | **W** | **F** | **D** | **T** | **N** | **W** | **K** |
| **0011** | **Plasma** | **4/8** | **S** | . | . | D | . | . | . | **T** |
|  |  | **2/8** | **S** | . | . | D | **S** | **K** | . | **T** |
|  |  | **1/8** | **S** | . | . | **S** | . | **K** | . | **T** |
|  |  | **1/8** | **S** | . | . | D | **S** | . | . | **T** |
|  | **LN DNA** | **3/10** | **S** | . | . | **S** | . | **K** | . | **T** |
|  |  | **3/10** | **S** | . | . | D | **S** | . | . | **T** |
|  |  | **3/10** | . | . | . | **S** | . | **K** | . | **N** |
|  |  | **1/10** | **S** | . | . | D | . | . | . | **T** |
|  | **LN RNA** | **2/5** | **S** | . | . | D | **S** | **K** | . | **T** |
|  |  | **2/5** | **S** | . | . | D | . | . | . | **T** |
|  |  | **1/5** | **S** | . | . | **S** | . | **K** | . | **T** |
|  | **PBMC DNA** | **3/10** | **S** | . | . | D | **S** | . | . | **T** |
|  |  | **2/10** | **S** | . | . | D | **S** | **K** | . | **T** |
|  |  | **2/10** | **S** | . | . | D | . | . | . | **T** |
|  |  | **2/10** | **S** | . | . | **S** | . | **K** | . | **T** |
|  |  | **1/10** | **S** | . | . | D | . | **K** | . | . |
|  | **PBMC RNA** | **6/10** | . | . | . | **S** | . | **K** | . | **N** |
|  |  | **3/10** | **S** | . | . | D | . | . | . | **T** |
|  |  | **1/10** | **S** | . | . | **S** | . | **K** | . | **T** |
| **0053** | **Plasma** | **8/9** | . | . | . | **S** | . | **E** | . | **R** |
|  |  | **1/9** | . | . | . | **S** | . | **E** | . | . |
|  | **LN** | **8/9** | . | . | . | **S** | . | **E** | . | **R** |
|  |  | **1/9** | . | . | . | **S** | . | **K** | . | . |
|  | **PBMC** | **5/10** | . | . | . | **S** | . | **K** | . | . |
|  |  | **4/10** | . | . | . | **S** | . | **E** | . | **R** |
|  |  | **1/10** | . | . | . | **S** | . | **K** | . | . |
| **079** | **Plasma** | **7/7** | **S** | . | . | D | . | . | . | **R** |
|  | **LN** | **7/8** | **S** | . | . | D | . | . | . | **R** |
|  |  | **1/8** | **S** | ***** | . | D | . | . | ***** | **R** |
|  | **PBMC** | **11/12** | **S** | . | . | D | . | . | . | **R** |
|  |  | **1/12** | **S** | . | . | D | . | . | . | . |
| **118** | **Plasma** | **8/8** | . | . | . | **N** | **S** | . | . | **Q** |
|  | **PBMC** | **5/9** | . | . | . | D | **S** | . | . | . |
|  |  | **3/9** | . | . | . | **N** | **S** | . | . | **Q** |
|  |  | **1/9** | . | . | . | **K** | **S** | . | . | . |
| **3011** | **Plasma** | **9/9** | . | . | . | **S** | . | . | . | . |
|  | **LN** | **6/8** | . | . | . | **S** | . | . | . | . |
|  |  | **1/8** | . | ***** | . | **S** | . | . | . | . |
|  |  | **1/8** | . | ***** | . | **S** | . | . | ***** | . |
|  | **PBMC** | **10/10** | . | . | . | **S** | . | . | . | . |
| **093** | **Plasma** | **10/10** | **S** | . | . | **S** | . | . | . | . |
|  | **LN** | **7/7** | **S** | . | . | **S** | . | . | . | . |
|  | **PBMC** | **7/9** | **S** | . | . | **S** | . | . | . | . |
|  |  | **2/9** | **S** | ***** | . | **S** | . | . | ***** | . |

**Supplementary Figure 14. bnAb escape mutations from epitopes in the MPER region.** Mutations that were different from the consensus C sequence (http://www.hiv.lanl.gov/) are shown in red font. Sites where the percentage of mutations present in one compartment is at least 50% different to another are shown in red blocks and expanded on in Table 3.5. Sequence frequency shows the number of times that specific sequence shows up in each compartment. HXB2 is the reference sequence that is used to number the amino acids in the participant alignments. Con C is the consensus C sequence from the HIV Los Alamos sequence database.
